# WNT signaling contributes to the extrahepatic bile duct proliferative response to obstruction in mice

**DOI:** 10.1172/jci.insight.181857

**Published:** 2024-12-05

**Authors:** Ashley N. Calder, Mirabelle Q. Peter, John W. Tobias, Nureen H. Mohamad Zaki, Theresa M. Keeley, Timothy L. Frankel, Linda C. Samuelson, Nataliya Razumilava

**Affiliations:** 1 Department of Internal Medicine, University of Michigan, Ann Arbor, Michigan, USA.; 2 Penn Genomics and Sequencing Core, Perelman School of Medicine, University of Pennsylvania, Philadelphia, Pennsylvania, USA.; 3 Department of Molecular and Integrative Physiology,; 4 Department of Surgery, and; 5 Rogel Cancer Center, University of Michigan, Ann Arbor, Michigan, USA.

**Keywords:** Hepatology, Cell cycle, Growth factors, Mouse models

## Abstract

Biliary obstruction and cholangiocyte hyperproliferation are important features of cholangiopathies affecting the large extrahepatic bile duct (EHBD). The mechanisms underlying obstruction-induced cholangiocyte proliferation in the EHBD remain poorly understood. Developmental pathways, including WNT signaling, are implicated in regulating injury responses in many tissues, including the liver. To investigate the contribution of WNT signaling to obstruction-induced cholangiocyte proliferation in the EHBD, we used complementary in vivo and in vitro models with pharmacologic interventions and transcriptomic analyses. To model obstruction, we used bile duct ligation (BDL) in mice. Human and mouse biliary organoids and mouse biliary explants were used to investigate the effects of WNT activation and inhibition in vitro. We observed an upregulation of WNT ligand expression associated with increased biliary proliferation following obstruction. Cholangiocytes were identified as both WNT ligand–expressing and WNT-responsive cells. Inhibition of WNT signaling decreased cholangiocyte proliferation in vivo and in vitro, while activation increased proliferation. WNT effects on cholangiocyte proliferation were β-catenin dependent, and we showed a direct effect of WNT7B on cholangiocyte growth. Our studies suggested that cholangiocyte-derived WNT ligands can activate WNT signaling to induce proliferation after obstructive injury. These findings implicate the WNT pathway in injury-induced cholangiocyte proliferation within the EHBD.

## Abstract 2

This study demonstrates that WNT signaling plays an important role in modulating the extrahepatic bile duct proliferative response to acute bile duct obstruction in mice.

## Introduction

Cholangiopathies are pathological processes that target the epithelium of the biliary tree (1). Due to a lack of effective interventions, cholestatic liver diseases, including cholangiopathies, account for 8% of liver transplants in the United States (2). They also substantially increase the risk of cholangiocarcinoma (CCA) development, a rare but highly aggressive form of cancer, that accounts for 2% of cancer-related death world-wide (3–5). Within the biliary tree, cholangiopathies show regional specificity, differing in incidence rates and outcomes between the intrahepatic bile duct (IHBD) and extrahepatic bile ducts (EHBD) (6–8). Thus, small-duct primary sclerosing cholangitis (PSC) has a slow rate of progression to fibrosis and cirrhosis and low risk of CCA. However, patients with large-duct PSC, including EHBD PSC, progress to cirrhosis quicker, requiring liver transplant or resulting in death within 9–20 years after diagnosis and developing biliary cancer in 10% of cases (9). These variances likely emerge from fundamental differences in developmental origin, cell composition, and anatomical location of IHBDs and the EHBD (10). This highlights the need to study these tissues independently. Previous research has primarily focused on the liver and IHBDs. Therefore, in this study, we focused on understanding the mechanisms regulating EHBD biology and the injury response.

Bile duct obstruction and cholangiocyte hyperproliferation are key features of biliary diseases (11–14). EHBD obstruction is observed in a diverse collection of biliary conditions including PSC, choledochal cyst, choledocholithiasis, and parasitic infections (8, 13). We previously showed that EHBD cholangiocytes, which are quiescent at homeostasis, rapidly respond to obstruction by proliferating to increase biliary mass (15). This proliferative response could serve as an initiating event for cholangiopathy progression and pathological processes when uncontrolled. Thus, understanding mechanisms and effects of this proliferative response to obstruction is relevant to promoting EHBD health and providing insight for new therapeutic approaches to mitigate cholangiopathy progression.

WNT signaling is a key regulator of epithelial cell proliferation and a modulator of stemness and differentiation within tissues of the gastrointestinal tract (16). It is composed of a complex network of 19 WNT ligands, 10 receptors, and several coreceptors that can activate downstream pathways via canonical (mediated by the transcription factor β-catenin) and noncanonical (β-catenin–independent) signaling. Canonical WNT signaling is commonly associated with cell proliferation and stem cell regulation (17, 18), while noncanonical WNT signaling has been implicated in cell polarity and migration (19). Furthermore, WNT signaling has been shown to have broad biological effects that are tissue and context specific. In the liver, WNT signaling is important for hepatic zonation and liver regeneration following injury (20–23). During development, WNT signaling is necessary for IHBD formation and drives progenitor cells toward a biliary cell fate (24). It has also been shown to participate in the regulation of cellular proliferation, transdifferentiation, and fibrosis in diseases affecting IHBDs (25–27). Both canonical and noncanonical WNT signaling are implicated in cholangiocyte proliferation within the IHBDs, while noncanonical WNT signaling has been shown to drive liver fibrosis progression (25, 27, 28). In contrast to the liver and IHBDs, the WNT signaling landscape in the EHBD are not well understood (29, 30). The limited studies thus far have shown that genetic overexpression of β-catenin in EHBD cholangiocytes induces biliary proliferation (30) and that canonical WNT signaling drives mesenchymal cell identity (31). However, whether WNT initiates cholangiocyte proliferation by directly targeting cholangiocytes and the cellular source of WNT ligands following EHBD obstruction are unknown.

In this study, we used in vivo mouse models, transcriptomics, morphometrics, and in vitro biliary models to define the WNT signaling landscape in the EHBD at homeostasis and in response to acute obstruction using a bile duct ligation (BDL) injury model. We demonstrated that the predominant WNT ligand (*Wnt7b*) and WNT target genes (*Birc5*, *Ccnd1*, *Myc*, and *Cd44*) (32–34) are present at homeostasis and are upregulated in cholangiocytes after acute EHBD injury. Inhibition of WNT ligand secretion reduced cholangiocyte proliferation in response to BDL. We further determined that cholangiocytes express WNT ligands based on our in vitro studies and transcriptomics analysis. Accordingly, in our in vitro biliary models, we observed opposing effects of WNT signaling activation and inhibition to enhance and reduce biliary proliferation, respectively. Lastly, we observed that cholangiocytes expressing WNT ligands can regulate their own proliferation via canonical (β-catenin–mediated) WNT signaling, and treatment with WNT7B increased organoid growth in vitro. These experiments provide a fundamental understanding of the WNT signaling landscape in the EHBD and establish cholangiocyte-initiated WNT signaling as an important regulator of biliary proliferation in the EHBD injury response.

## Results

### Acute obstructive bile duct injury is associated with an upregulation of cell cycle genes and epithelial cell proliferation in the EHBD.

**Figure 1:**
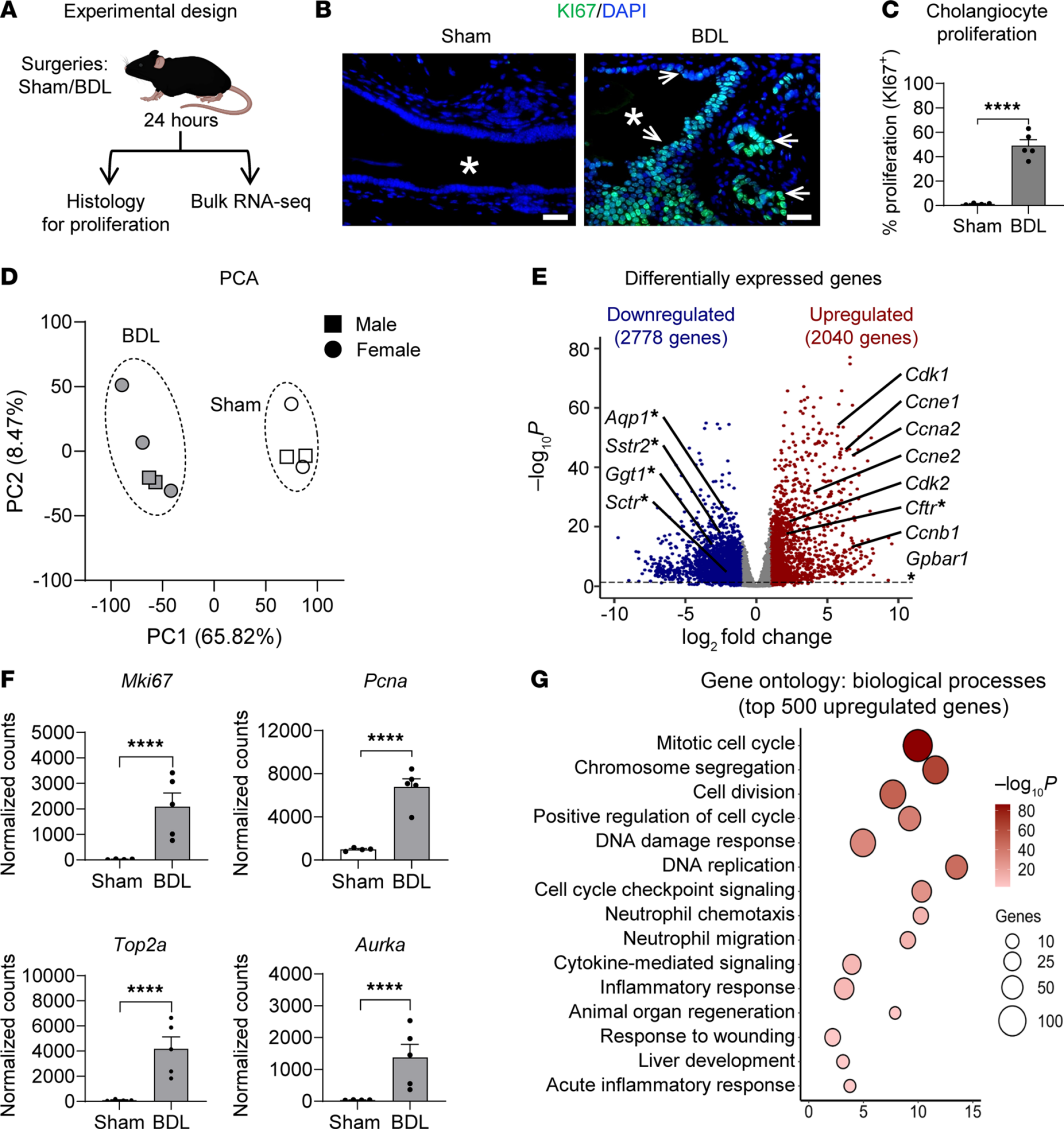
Acute injury induces epithelial cell proliferation and upregulation of cell cycle–related genes. (**A**) Schematic of sample collection for histological and transcriptomic assessment of the EHBD after BDL. (**B**) Ki67 (green) and DAPI (blue) immunofluorescence staining 24 hours after BDL. Asterisks indicate lumen, arrows indicate Ki67^+^ cells. Scale bar: 20 μm. (**C**) Morphometric analysis of cholangiocyte proliferation in sham/BDL EHBDs. (**D**) Principal component analysis (PCA) from bulk RNA-Seq of sham/BDL EHBDs 24 hours after surgery. (**E**) Volcano plot depicting DEGs, cyclins, and their kinases associated with cell cycle as well as functional cholangiocyte genes (indicated by asterisks). (**F**) Normalized counts for proliferation markers *Pcna*, *Mki67*, *Top2a*, and *Aurka* from sham/BDL EHBDs. (**G**) Ontology of the top 500 upregulated genes from sham/BDL EHBDs. Unpaired Student’s *t* test for morphometric analysis of proliferation. Statistical significance of bulk RNA-Seq was assessed using DESeq2. ****P < 0.0001. *n* = 4–5 mice/group.

Cholangiocyte hyperproliferation is a key feature throughout the spectrum of biliary diseases, from acute obstruction to carcinogenesis (5, 15, 35). We have previously shown that acute obstruction induced by BDL activates cholangiocyte proliferation in the EHBD that surges at 24 hours and largely subsides by 48 hours, which involves a complex cellular crosstalk under the control of Hedgehog (HH) signaling through the recruitment of immune cells (15). However, the other mechanisms underlying this proliferative transformation are incompletely understood. To define the molecular response to BDL, we conducted histological and transcriptional analysis of the bile duct at 24 (Figure 1A) and 48 hours to gain insight into the dynamic changes.

We verified a rapid cholangiocyte proliferative response by staining for the proliferation marker Ki67 at 24 hours after BDL (Figure 1, B and C) (15). Transcriptomic analysis of cell senescence gene *Cdkn1a* (encodes the P21 protein) showed an upregulation 24 hours after BDL (Supplemental Figure 1A; supplemental material available online with this article; https://doi.org/10.1172/jci.insight.181857DS1) while immunofluorescence staining localized P21 primarily to the stromal cell compartment (Supplemental Figure 1B). We also observed little to no apoptosis in sham or BDL samples as assessed by Cleaved Caspase-3 immunofluorescence staining at 24 hours after surgery (Supplemental Figure 1C).

To assess the transcriptional changes to the EHBD in acute injury, we performed bulk RNA-Seq analysis of the EHBDs at 24 hours after surgery (Figure 1, D–G). Principal component analysis (PCA) demonstrated clustering of sham and BDL samples, indicating that surgical treatment was the highest contributor to differences among the samples (Figure 1D). We did not observe any apparent sex differences in either sham or BDL groups based on PCA. BDL induced a robust change in the EHBD transcriptional profile with over 4,500 differentially expressed genes (DEGs) with a fold change > 2 (Figure 1E).

Cyclins and their associated kinases necessary for the control of cell cycle progression were among the most highly upregulated genes following 24-hour BDL. Furthermore, other cell cycle–related genes, including *Mki67*, *Pcna*, *Top2a*, and *Aurka*, were highly expressed after BDL (Figure 1F). In accordance with increased cellular proliferation, we observed a downregulation of functional cholangiocyte markers *Aqp1*, *Sstr2*, *Ggt1*, and *Sctr* (Figure 1E). To determine cellular processes and pathways enriched following 24-hour BDL, we performed an ontology enrichment analysis of the top 500 upregulated genes using Metascape (a gene annotation and analysis resource) (36). This gene ontology analysis demonstrated enrichment in terms related to cell cycle, immune cell regulation, and inflammatory responses (Figure 1G), with a complete list of enriched processes for both up- and downregulated genes included in Supplemental Table 1.

To examine later EHBD changes after obstructive injury, we extended the duration of the sham and BDL surgeries. We had to limit the duration of the experiment to 48 hours as the mice did not tolerate distal BDL beyond 72 hours, with unacceptably high mortality rates likely due to gallbladder rupture (Supplemental Figure 2). Therefore, we conducted bulk RNA-Seq analysis on EHBDs from sham/BDL mice at 48 hours. Similar to our 24-hour dataset, we observed clustering of sham and BDL groups in a PCA plot from our bulk RNA-Seq dataset at 48 hours and observed no obvious sex differences (Supplemental Figure 3).

Proliferation markers *Mki67*, *Pcna*, *Top2a*, and *Aurka* remained elevated 48 hours after BDL (Supplemental Figure 4A). This supports our previous findings that, while cholangiocyte proliferation peaks at 24 hours and largely subsides at 48 hours, a delayed stromal proliferative response occurs at 48 hours and is likely responsible for the still-elevated expression of proliferation markers at this time point (15).

As cholangiopathies are often associated with a proinflammatory and profibrotic phenotype, we analyzed both the 24- and 48-hour postsurgery transcriptomic datasets for genes encoding inflammatory cytokines and extracellular matrix–related (ECM-related) proteins. We observed an increase in several inflammatory cytokines, including IL-1β (*Il1b*), IL-6 (*Il6)*, and TNF-α (*Tnfa*) after BDL (Supplemental Figure 4B). Furthermore, many genes of ECM-related proteins, including collagens, matrix metalloproteinases (MMPs), tissue inhibitors of metalloproteinases (TIMPs), hyaluronic acid (HAS), and TGF-β genes, were dysregulated at 48 hours after injury (Supplemental Figure 5). This suggested potential remodeling of the ECM in response to bile duct obstruction.

Lastly, to determine the effect of BDL on serum markers of cholestasis and obstruction, we conducted liver function tests. We observed a significant increase in alkaline phosphatase (ALP), a classic marker of cholestasis at 48 hours after BDL, and total bilirubin (TBILI), a marker of obstruction, at 24 and 48 hours after BDL. Additionally, we saw increases in aspartate aminotransferase (AST) and alanine transaminase (ALT), markers of hepatocyte injury (Supplemental Figure 6) at 48 hours after BDL, indicating effective obstruction and liver injury at the later time point.

These data verify a robust proliferative response in cholangiocytes, accompanied by transcriptional remodeling in the EHBD to drive proliferation. Accordingly, we focused on the mechanisms driving this acute cholangiocyte proliferative response to obstruction in the EHBD 24 hours after BDL.

### WNT signaling is upregulated in the EHBD following BDL.

Activation of developmental pathways after injury is known to contribute to cell proliferation. WNT signaling plays a vital role in the regulation of epithelial cell biology in the gastrointestinal tract and liver at homeostasis and is upregulated in proproliferative models of cholestasis and CCA within the IHBDs (16, 20, 25, 26, 28, 37). We focused further analysis on our 24-hour time point, as this is when we observed peak cholangiocyte proliferation previously. We first assessed the expression of the known 19 WNT ligands at homeostasis (sham mice) and showed that *Wnt4*, *Wnt5a*, *Wnt7a*, and *Wnt7b* are the most abundant ligands in the EHBD, with *Wnt7b* being the most prevalent (Supplemental Figure 7A). We further observed baseline expression of several WNT receptors, *Frizzled* (*Fzd*), with *Fzd1* and *Fzd4* being the predominant receptors at homeostasis (Supplemental Figure 7B). R-spondin ligands (RSPO) bind to their receptors, leucine-rich repeat-containing G protein–coupled receptor (LGR), to function as enhancers of WNT signaling by stabilization of the FZD receptor. We observed the expression of ligands *Rspo1*, *Rspo3*, and *Lgr4* receptor primarily at homeostasis (Supplemental Figure 7C).

**Figure 2:**
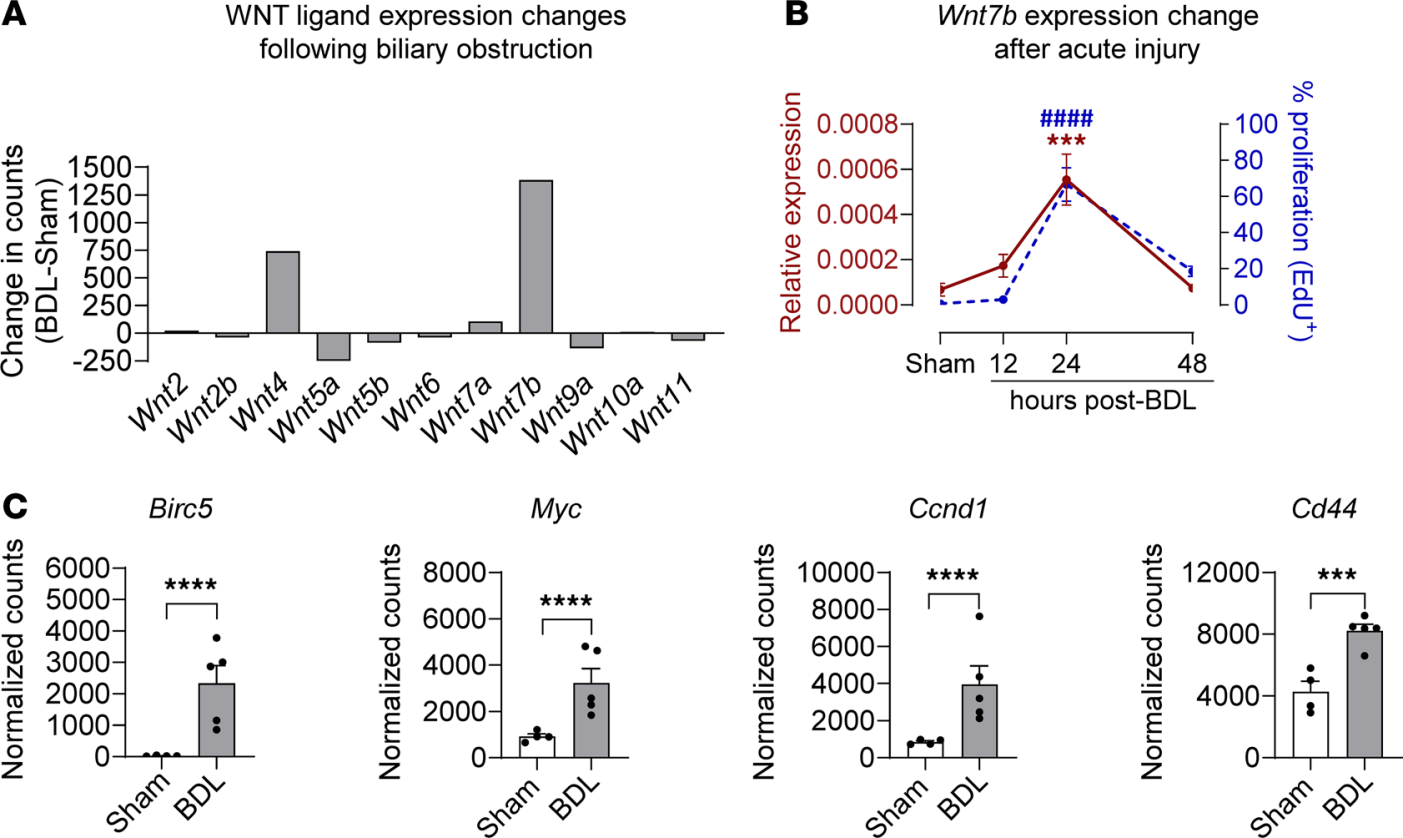
WNT signaling is upregulated following 24-hour EHBD injury. (**A**) Bulk RNA-Seq analysis of transcription differences among the WNT ligands expressed in the EHBD. (**B**) qPCR of the EHBD dominant WNT ligand *Wnt7b* (left *y* axis) and cholangiocyte proliferation (right *y* axis) in sham (24 hour) and ligated mice at 12, 24, and 48 hours after BDL (15). (**C**) Bulk RNA-Seq analysis of WNT target gene expression changes after BDL. One-way ANOVA with Bonferroni’s multiple-comparison test was used to assess qPCR data. Statistical significance of targets identified by the bulk RNA-Seq analysis was assessed using DESeq2. ***P < 0.001, ****P < 0.0001, ^####^*P* < 0.0001, 1-way ANOVA with Bonferroni’s multiple-comparison test was used to assess proliferation rates. *n* = 3–13 mice/group.

Furthermore, we examined changes in expression levels of WNT signaling components after injury (24 hours after BDL) in our RNA-Seq dataset. Most notably, there was an increase in *Wnt7b* abundance, suggesting that WNT signaling and specifically *Wnt7b* may contribute to the observed proliferative response (Figure 2A). Our previous investigation has shown that cholangiocyte proliferation in the EHBD is not significantly increased at 12 hours, peaks at 24 hours, and largely subsides by 48 hours after injury (15). To determine if WNT ligand expression correlates with the temporal dynamics of the cholangiocyte proliferative response, we measured EHBD *Wnt7b* mRNA expression using quantitative PCR (qPCR) at 12, 24, and 48 hours after BDL. We found that *Wnt7b* abundance is temporally correlated with the cholangiocyte proliferative response, further suggesting that WNT ligands may play a role in the regulation of biliary proliferation (Figure 2B) (15). We also observed significant increases in the abundance of WNT target genes (*Birc5*, *Myc*, *Ccnd1*, and *Cd44*) implicated in cell proliferation and survival in our bulk RNA-Seq dataset (Figure 2C) (38–41). Lastly, we observed differential expression of several low-expressing WNT ligands, *Rspo1* and *Rspo3*, and several *Fzd* receptors (Supplemental Figure 7, D–F) 24 hours after BDL. Together, these data suggest that acute obstructive EHBD injury leads to changes in WNT signaling and may closely correspond to the timeline of the cholangiocyte proliferative response.

### Cholangiocytes are WNT responsive and secrete WNT ligands in the EHBD at baseline and after injury.

**Figure 3:**
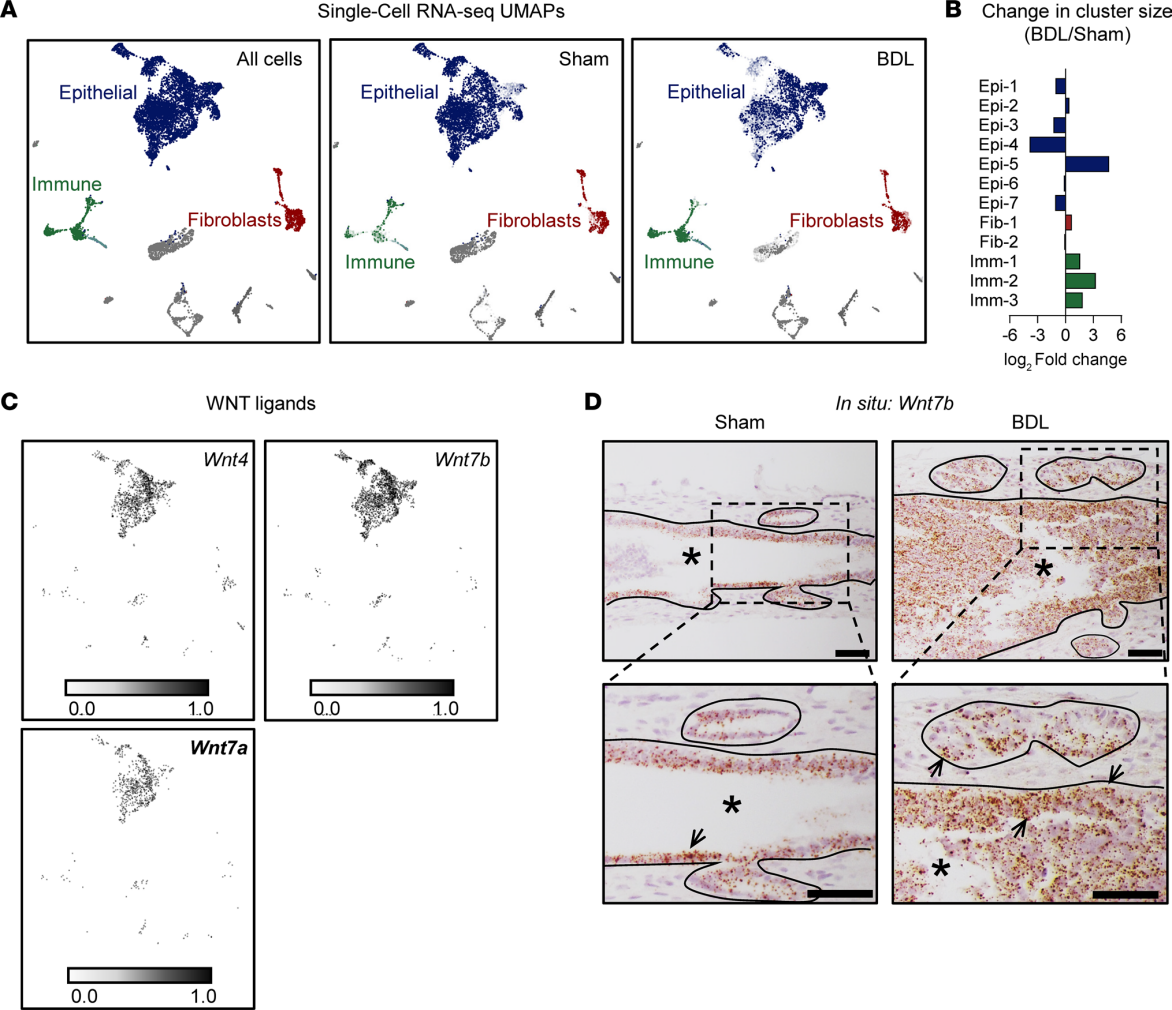
Cholangiocytes express WNT ligands. (**A**) UMAP with epithelial (blue), fibroblast (red), and immune (green) cell clusters shown. Distribution of cells from the combined sham/BDL samples (left), sham alone (middle), and BDL (right). (**B**) Changes in cluster compositions following BDL based on cluster proportions. Epi, epithelial; Fib, fibroblast; Imm, immune cell clusters. (**C**) Expression of the predominant WNT ligands in the EHBD. *n* = 2 samples/treatment (1 male, 1 female), *n* = 5 mice/sample. (**D**) In situ hybridization for *Wnt7b* in the EHBD of sham/BDL mice. Asterisks indicate lumen, line separates epithelial and stromal cell compartments, lower panels show magnification of area indicated by dashed black box, and arrows indicate Wnt7b*^+^* cells. Scale bar: 50 μm. *n* = 3 mice/group.

WNT signaling often facilitates communication between different cell types to regulate tissue-wide responses. Accordingly, we next aimed to determine target cells for WNT signaling in the EHBD at homeostasis and after acute injury by conducting single-cell RNA-Seq (scRNA-Seq) analysis using the BDL injury model. Our dataset contained 3,696 and 5,891 cells in the EHBD 24-hours post-BDL and sham groups, respectively. We observed 21 unique cell clusters, which we characterized using the top DEGs (Supplemental Figure 8). Of these, we focused on cell clusters pertaining to the 3 main cell types found within the EHBD: epithelial (*Epcam*, *Krt19*, and *Prom1*), stromal fibroblasts (*Col1a1*, *Col1a2*, *Col5a1*), and immune (*Ptprc*, *Itgam*, and *Itgb2*) cell clusters (Figure 3A). Within these clusters, epithelial cells (77%) made up the majority of the cells analyzed with a similar expression of fibroblast (11%) and immune (12%) cell populations in the combined surgical groups. Notably, we observed an expansion of immune clusters following BDL (22%) compared with sham mice (6%) (Figure 3B), supporting our previous findings demonstrating immune infiltration in the EHBD following BDL (15).

We next sought to determine the cellular source of WNT ligands. Accordingly, we analyzed our scRNA-Seq dataset for the predominant EHBD WNT ligands and mapped *Wnt4*, *Wnt7a*, and *Wnt7b* primarily to cholangiocyte clusters (Figure 3C). *Wnt5a* expression was too low to definitively localize. We performed in situ hybridization (ISH) for *Wnt7b* and confirmed primarily cholangiocyte localization of *Wnt7b* in both sham and BDL EHBDs (Figure 3D).

**Figure 4:**
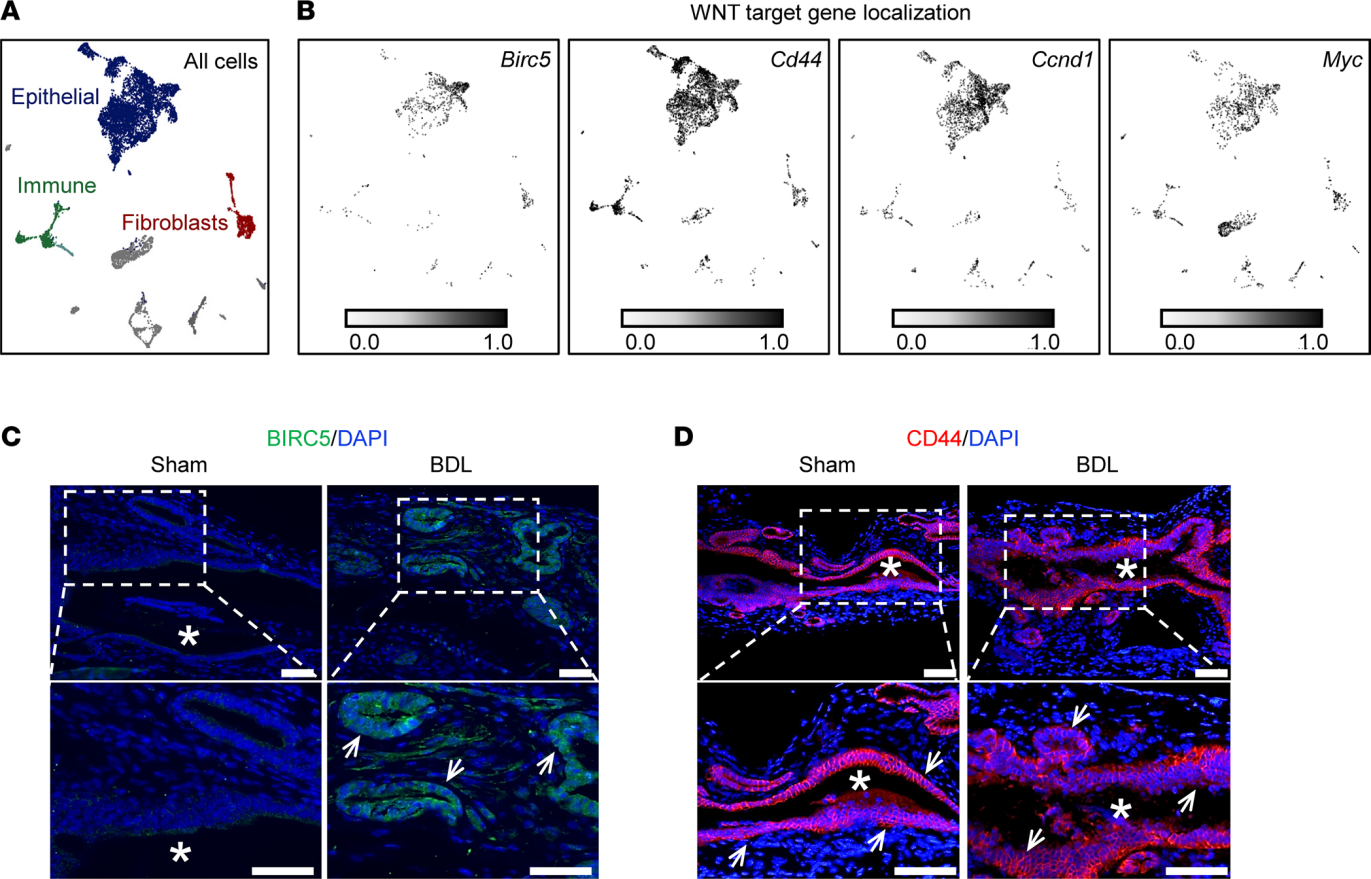
Cholangiocytes are WNT target cells. (**A**) UMAP of combined cells from sham/BDL scRNA-Seq datasets. (**B**) WNT target genes localize primarily to epithelial cells clusters in all scRNA-Seq samples. *n* = 2 samples/treatment (1 male, 1 female), 5 mice/sample. (**C** and **D**) BIRC5 (green) and CD44 (red) immunofluorescence staining with DAPI (blue) in sham/BDL mice. Asterisks indicate lumen, lower panels show increased magnification of dotted white box, arrows indicate BIRC5^+^ and CD44^+^ cells, respectively. Scale bar: 50 μM. *n* = 3 mice/group.

To determine if cholangiocytes are WNT target cells, we assessed the localization of upregulated WNT target genes *Birc5*, *Cd44*, *Ccnd1*, and *Myc*. These genes primarily mapped to epithelial cell clusters in EHBDs of both sham and BDL mice (Figure 4, A and B). We further verified WNT target protein expression in epithelial cells with BIRC5 and CD44 immunofluorescence in sham and BDL mice (Figure 4, C and D). These data suggest that epithelial cells express WNT ligands and are targets of WNT signaling.

### Canonical WNT signaling contributes to the regulation of mouse and human EHBD cholangiocyte proliferation.

**Figure 5:**
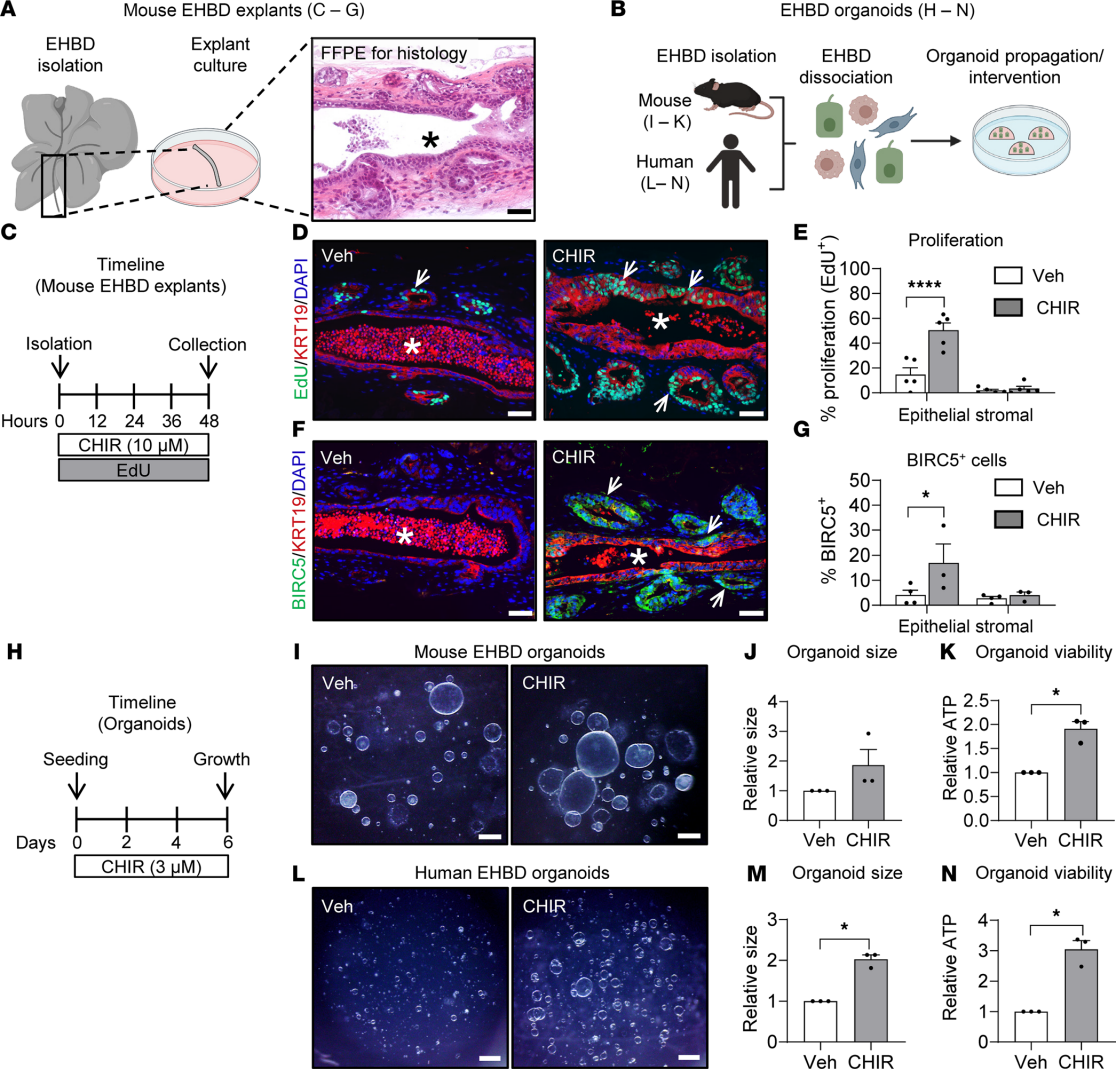
Canonical WNT activation promotes cholangiocyte proliferation in mouse EHBD explants and mouse and human EHBD organoids. (**A** and **B**) Diagram of EHBD explant and organoid culturing protocols. (**C**) Timeline of EHBD explant experiment with 10 μM CHIR treatment. (**D**) EdU (green), KRT19 (red), and DAPI (blue) staining in explants. (**E**) Morphometric analysis of proliferation in biliary explants. (**F** and **G**) BIRC5 (green), KRT19 (red), and DAPI (blue) staining in explants and quantification. Asterisks indicate lumen, arrows indicate EdU^+^ and BIRC5^+^ cells. Scale bar: 50 μm. *n* = 3–5 mice/group. (**H**) Timeline of the experiment treating mouse and human biliary organoids with 3 μM CHIR. (**I**–**K**) Mouse organoid images (**I**), size (**J**), and viability (**K**) by measurement of culture ATP levels in vehicle- and CHIR-treated organoids.(**L**–**N**) Human organoid images (**L**), size (**M**), and viability (**N**) measurements in vehicle- and CHIR-treated organoids. *n* = 3 biological replicates. Scale bar: 500 μm. Size and viability measurements are normalized the vehicle controls. Two-way ANOVA with Bonferroni’s multiple-comparison test was used for morphometric analysis of EdU and BIRC5. Unpaired Student’s *t* test was used for organoid experiments. *P < 0.05, ****P < 0.0001.

Our transcriptomic datasets suggest that EHBD cholangiocytes can be both WNT ligand–secreting and WNT-responsive cells. To directly assess the effects of WNT signaling activation on cholangiocyte proliferation, we used in vitro mouse EHBD explant (Figure 5A) and EHBD organoid (Figure 5B) models. Mouse EHBD explants have low basal proliferation, similar to EHBD tissue under homeostasis (Figure 5D). We induced WNT signaling in explants by treatment with CHIR-99021 (CHIR), a glycogen synthase kinase 3 (GSKβ) inhibitor that stabilizes β-catenin and, therefore, stimulates canonical WNT signaling (Figure 5C). Evaluation for the cholangiocyte marker keratin 19 (KRT19) and the proliferation marker EdU demonstrated that stimulation of canonical WNT signaling with CHIR increased proliferation in the EHBD cholangiocytes (Figure 5, D and E). We also observed increased expression of the WNT target BIRC5 in cholangiocytes of the CHIR-treated explants (Figure 5, F and G), findings similar to our in vivo observations after BDL (Figure 2C).

Next, we examined the epithelium-specific effects of canonical WNT stimulation with CHIR on cholangiocytes using a reductionist EHBD organoid model derived from mouse and human EHBD tissue (Figure 5H). These 3D structures are composed of biliary epithelial cells that allow the direct study of WNT signaling on cholangiocytes (42). Despite cholangiocytes in organoids already exhibiting a proliferative state (43), we observed a further increase in organoid growth after WNT stimulation in mouse (Figure 5, I–K) and human EHBD organoids (Figure 5, L–N). These data indicate that EHBD cholangiocyte proliferation is directly activated by canonical WNT signaling.

### Cholangiocyte-derived WNT ligands activate canonical WNT signaling to stimulate growth.

**Figure 6:**
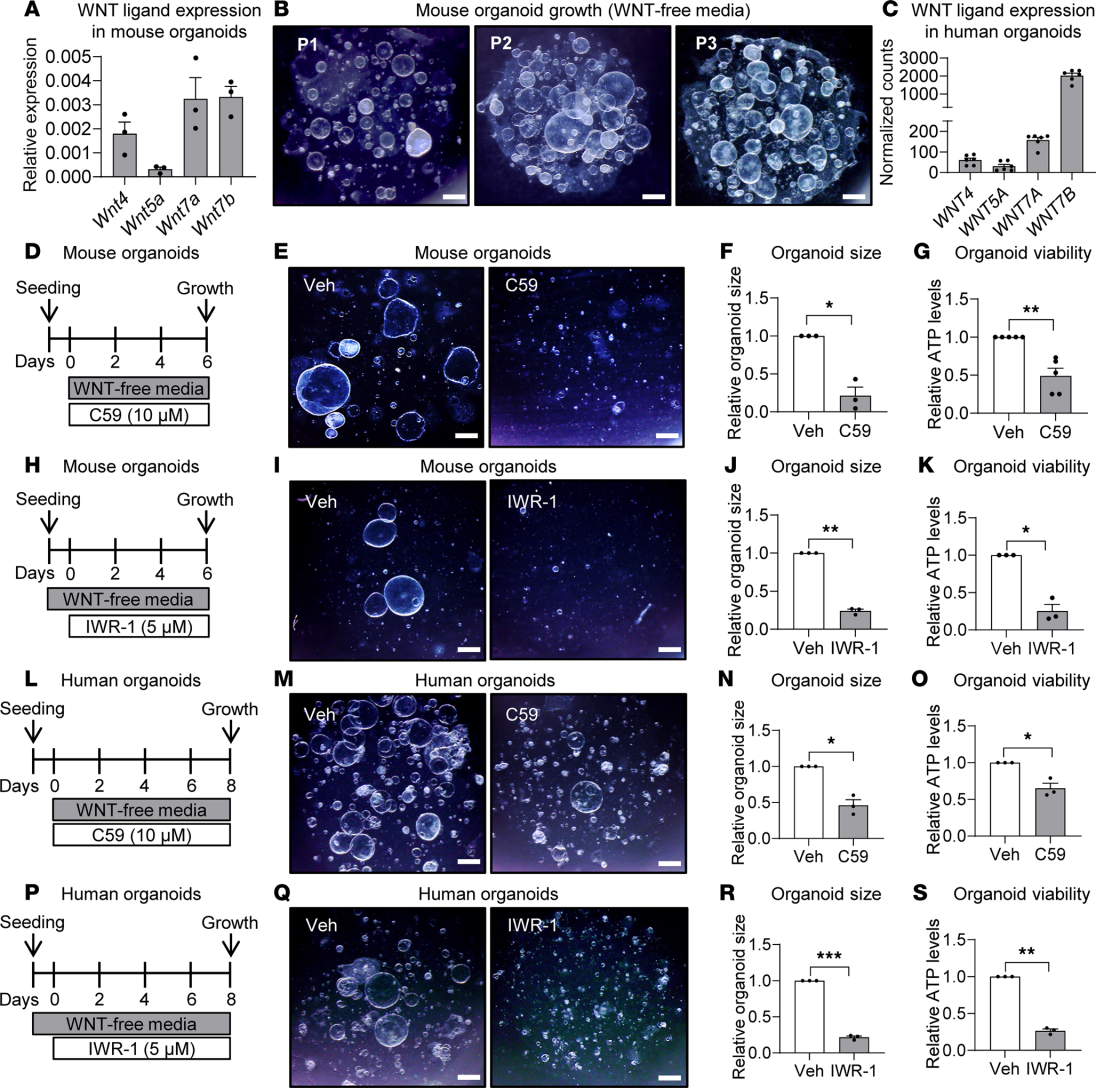
Endogenous WNT ligands promote organoid growth through activation of canonical WNT signaling. (**A**) qPCR from mouse EHBD organoids for the 4 most abundant WNT ligands of the EHBD. (**B**) Images from mouse organoid cultures 1, 2, and 3 passages after being grown in WNT-free media. (**C**) Bulk RNA-Seq analysis of human organoids for WNT ligands. (**D**) Timeline for C59 administration to inhibit WNT secretion by mouse organoids. (**E**–**G**) Mouse organoid images (**E**), size (**F**), and viability (**G**) measurements after treatment with 10 μM C59. (**H**) Timeline for IWR-1 administration to inhibit β-catenin in mouse organoids. (**I**–**K**) Mouse organoid images (**I**), size (**J**), and viability (**K**) following 5 μM IWR-1 treatment. (**L**) Timeline for C59 administration in human organoids. (**M**–**O**) Human organoid images (**M**), size (**N**), viability (**O**) after 10 μM C59 treatment. (**P**) Timeline for IWR-1 treatment in human organoids. (**Q**–**S**) Human organoid images (**Q**), size (**R**), and viability (**S**) following 5 μM IWR-1 treatment. Size and viability measurements are normalized to the vehicle controls. Scale bar: 500 μm. One-sample *t* test. *P < 0.05, **P < 0.01, ***P < 0.001, *n* = 3–5 biological replicates.

Since we localized both WNT ligands and target genes to EHBD cholangiocytes after injury (Figures 3 and 4), we further dissected the direct effects of cholangiocyte-derived WNT ligands on biliary proliferation in EHBD organoids. Using qPCR, we verified the expression of *Wnt4*, *Wnt5a*, *Wnt7a*, and *Wnt7b* in mouse organoids (Figure 6A). We assessed WNT ligands, receptors, and targets expressed in human EHBD biliary cells by analyzing a published bulk RNA-Seq dataset on human EHBD organoids (E-MTAB-7569) (44) (Supplemental Figure 9). *WNT7B* had the greatest RNA abundance among ligands (Figure 6C), suggesting that human EHBD cholangiocytes are WNT ligand–expressing and WNT-responsive cells similar to mouse EHBD cholangiocytes.

Cholangiopathies in patients are associated with biliary hyperproliferation and WNT signaling dysregulation (7, 45). Our analysis of The Cancer Genome Atlas (TCGA) RNA-Seq datasets from CCA and control tissues showed an increase in proliferation markers *Ki67* and *PCNA* in CCA (Supplemental Figure 10A). Additionally, in CCA samples, *WNT7B* expression and expression of WNT targets *BIRC5* and *CD44* were significantly increased as compared with control tissues (Supplemental Figure 10, B and C), indicating that WNT signaling may play a role in human biliary cancer biology.

Notably, the standard EHBD organoid culture medium contains WNT activators such WNT3A and RSPO3 ligands (42, 44, 46). To assess if EHBD organoid cultures could sustain growth by relying only on endogenous WNT ligands, we cultured mouse organoids over the course of 3 passages (21 days in culture) without medium supplementation with WNT3A and RSPO3 ligands (Figure 6B). Notably, organoid cultures were able to maintain growth and a cystic morphology similar to that seen when cultured with exogenous WNT supplementation. Thus, we showed that cholangiocyte organoids express WNT ligands and can grow without exogenous WNT supplementation in culture.

We next aimed to determine if cholangiocyte-derived WNT ligands are necessary to support organoid growth. We treated mouse organoids cultured in WNT-free media with C59, which prevents WNT ligand secretion from cells via inhibition of PORCN (an enzyme necessary for WNT ligand palmitoylation and secretion from the cells) (47) (Figure 6D). Organoids treated with C59 showed a significant decrease in size (Figure 6, E and F) and viability (Figure 6G) as compared with vehicle-treated cultures. Furthermore, we were able to recover the loss in organoid growth due to C59 by activation of canonical WNT signaling via CHIR (Supplemental Figure 11A), confirming that these effects are WNT mediated.

*Wnt7b* was the most transcriptionally abundant WNT ligand in the EHBD, and it was upregulated after BDL. We assessed the proliferative effect of WNT7B on mouse biliary organoids using L cell conditioned media expressing *Wnt7b* (Supplemental Figure 12A). Culturing mouse organoids with WNT7B conditioned media increased growth and organoid size compared with organoids treated with control L cell media (Supplemental Figure 12, B–D). Furthermore, treatment with WNT7B increased expression of WNT target genes *Myc*, *Ccnd1*, and *Cd44* in mouse organoid lines (Supplemental Figure 12E). These data demonstrate a direct proliferative effect of WNT7B on EHBD cholangiocytes in vitro, consistent with the effects we observed post-BDL in vivo.

Since our RNA-Seq analysis showed an upregulation of canonical WNT signaling target genes (Figure 2C) that localized to cholangiocytes (Figure 4, B–D), we hypothesized that WNT ligands mediate canonical WNT signaling in organoids to promote their growth. Accordingly, we treated mouse organoids with WNT antagonist 1 (IWR-1), which stabilizes the destruction complex and thereby inhibits β-catenin and canonical WNT signaling (48) (Figure 6H). As expected, we observed a decrease in organoid size (Figure 6, I and J) and viability (Figure 6K) following IWR-1 treatment. Last, to determine if the WNT signaling effects observed in mouse EHBD cholangiocytes are applicable to human EHBD cholangiocytes, we conducted experiments with C59, IWR-1, and CHIR in human EHBD organoid cultures. We observed decreases in human organoid growth and viability upon inhibition of WNT ligands (Figure 6, L–O), of which the former could be rescued with canonical WNT activation (via CHIR) (Supplemental Figure 11B), and similar decreases in organoid size and viability with inhibition of β-catenin signaling (Figure 6, P–S). Together, these experiments suggested that both mouse and human EHBD cholangiocyte-derived WNT ligands can activate canonical WNT signaling to maintain organoid growth and that WNT7B has a direct proliferative effect on EHBD cholangiocytes.

### WNT signaling regulates EHBD cholangiocyte proliferation.

**Figure 7:**
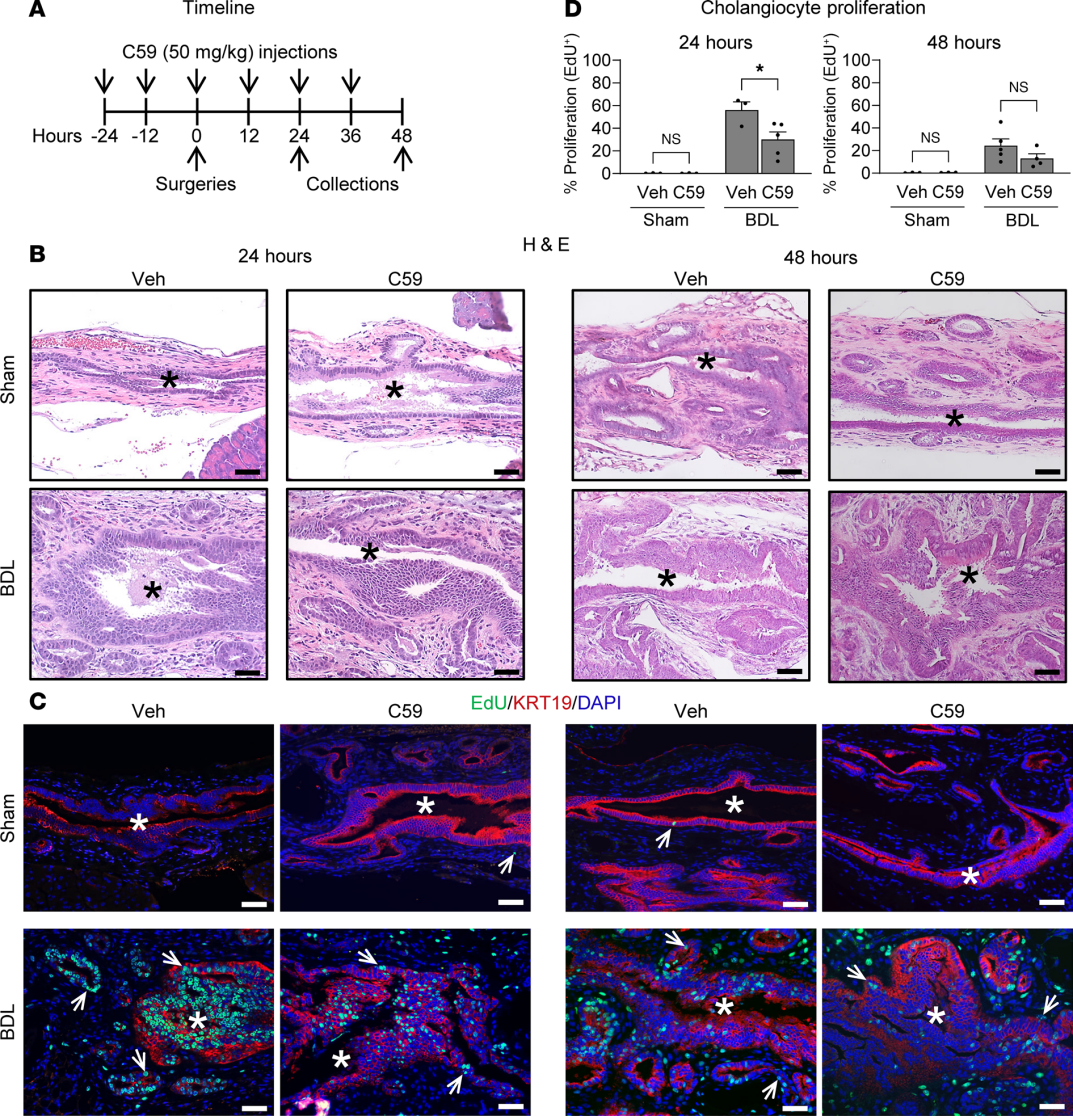
Inhibition of WNT ligand secretion leads to decreased cholangiocyte proliferation in the injured mouse EHBD. (**A**) Timeline of C59 administration during sham and BDL surgeries. (**B**) H&E staining of EHBDs of sham/BDL mice treated with either vehicle (Veh) or C59. (**C**) EdU (green), KRT19 (red), and DAPI (blue) immunofluorescence staining of Veh/C59-treated sham/BDL mice. (**D**) Morphometric analysis of cholangiocyte proliferation using EdU marker. Asterisks indicate lumen, and arrows indicate EdU^+^ cholangiocytes. Scale bar: 50 μm. Two-way ANOVA was used to compare sham and BDL samples with Bonferroni’s multiple-comparison test. *P < 0.05. *n* = 3–5 mice/group.

In our mouse EHBD injury model, we observed that upregulation of WNT ligands (Figure 2, A and B) is associated with increased biliary proliferation (Figure 1C). We also observed that WNT ligands (including WNT7B) and canonical WNT signaling can modulate cholangiocyte proliferation to induce organoid expansion (Supplemental Figure 12, B–D, and Figure 6). Therefore, to determine if WNT ligands play a functional role in BDL-induced cholangiocyte proliferation in vivo, we treated mice undergoing sham/BDL surgeries with C59 to inhibit WNT ligand secretion (Figure 7A). We observed no gross histological changes in C59-treated EHBDs at 24 or 48 hours after BDL when compared with their vehicle controls, based on H&E examination (Figure 7B). However, there was a significant reduction in cholangiocyte proliferation in the BDL mice treated with C59 compared with BDL mice treated with vehicle at 24 hours after BDL (Figure 7, C and D). These data suggest that WNT ligands are involved in promoting cholangiocyte proliferation following obstructive EHBD injury in mice.

Since we observed changes in inflammatory markers and ECM-related genes 24 hours after BDL, we assessed their status upon WNT inhibition. We found no significant change in *Il1b*, *Il6*, or *Tnfa* after C59 treatment at this time point (Supplemental Figure 13). Furthermore, since we observed dysregulation of ECM-related genes following BDL (Supplemental Figure 5), and as fibrosis is an important component of cholangiopathies, we examined EHBDs from vehicle- or C59-treated (Veh/C59-treated) mice for collagen expression using Masson’s trichrome staining. We found no changes in collagen staining between sham and BDL groups or upon WNT inhibition at 24 or 48 hours after BDL (Supplemental Figure 14), suggesting collagen deposition as a later phenotype occurring in cholangiopathies and not influenced by WNT signaling during early response to obstruction.

We next assessed the effects on WNT inhibition (via C59) on cholestasis by conducting liver function tests on serum collected from Veh/C59 BDL mice 48 hours after obstruction. We saw no changes in markers of cholestasis, obstruction, or hepatocyte injury at this time point (Supplemental Figure 15). These data suggest that, while we demonstrate an increase in markers of liver injury at 48 hours after BDL, C59 did not reduce liver injury at this acute time point. This finding supports a previous study that found no effect of C59 treatment on liver function tests at 7 days after BDL (49).

## Discussion

Regulation of tissue homeostasis and injury responses are important to promote organ health and mitigate the consequences of maladaptive responses. Bile duct obstruction often accompanies large bile duct cholangiopathies and is associated with increased biliary proliferation (7). In the liver, biliary proliferation can facilitate recovery of organ function by replenishing depleted cholangiocyte populations. However, it can also have maladaptive consequences in the form of ductular reaction associated with a proinflammatory, profibrotic, and hyperproliferative cholangiocyte phenotype, which can worsen fibrosis and result in cancer development (7, 50, 51). Although this sequence has been established in the liver, the mechanisms responsible for the EHBD proliferative response to biliary obstruction are poorly understood. We have previously identified HH signaling as a regulator of neutrophil influx in the injured EHBD contributing to biliary hyperproliferation and showed that HH can cooperate with IL-33 to induce hyperproliferation (15, 52). In this study, we provide evidence for the participation of WNT signaling in the cholangiocyte proliferative response to injury. We determined that WNT target genes that promote cell proliferation and survival are rapidly upregulated after obstructive injury along with WNT ligand *Wnt7b*. Using a set of functional in vivo and in vitro studies in mouse and human samples, we demonstrated that canonical WNT signaling in cholangiocytes can be initiated in an autocrine manner to promote biliary proliferation. Therefore, we provide knowledge about WNT changes accompanying EHBD injury, suggesting that accentuated WNT signaling contributes to initiation of cholangiocyte proliferation, after biliary obstruction, and that cholangiocyte-derived WNT ligands can promote their own β-catenin–dependent signaling.

Within the IHBD, WNT signaling is involved in many aspects of biliary disease and cholangiocyte proliferation in a highly context-dependent manner (25, 26). However, its role in EHBD injury response has remained largely unexplored. We observed a rapid increase in proliferation markers in cholangiocytes of the EHBD with a parallel increase in *Wnt7b* expression at 24 hours after BDL, with both largely subsiding by 48 hours. This acute post-BDL upregulation of *Wnt7b* was accompanied by an increase in WNT target genes *Birc5*, *Ccnd1*, and *Myc*. We further demonstrated that WNT7B induces growth and WNT target gene expression in primary cholangiocytes using our organoid system. These data support previous findings that overexpression of *Wnt7b* in an immortalized small cell cholangiocyte cell line (SMCC), characteristic for IHBD cholangiocytes, increases cholangiocyte proliferation (28). Thus, while the increase in WNT7B after BDL is likely involved in initiating the cholangiocyte proliferative response, it is also possible that other predominant WNT ligands (WNT4, WNT5A, WNT7A) participate in regulation of proliferation. Previous studies on IHBDs have implicated both canonical and noncanonical WNT signaling in models of biliary cholestasis and CCA (25–28). Accordingly, using EHBD explants, we showed that cholangiocytes are the only cell population to rapidly proliferate in response to activation of canonical WNT signaling in the EHBD. Furthermore, mouse and human cholangiocytes express all 4 of the predominant WNT ligands in vitro, and we show that their effects on cholangiocyte growth are β-catenin mediated. Lastly, our data show that the inhibition of WNT ligand secretion in mice using C59 suppressed the EHBD cholangiocyte proliferative response. Since cholangiocytes appear to be a predominant source of WNT ligands after BDL, this suggests that canonical WNT signaling in EHBD cholangiocytes contributes to their proliferative response to obstructive injury. Our work will provide a foundation for future studies exploring the role of WNT signaling in the EHBD injury response and further delineating the role of canonical and noncanonical WNT signaling in the context of biliary disease.

When we inhibited WNT signaling during mouse EHBD injury response by suppressing WNT ligand secretion with C59 in vivo, we showed a significant reduction in cholangiocyte proliferation after injury. These effects mirror those seen previously with HH inhibition following 24-hour BDL in the EHBD as described by Zaki et al. (15). This suggests possible cooperation of these pathways in EHBD injury responses. Within the EHBD, loss of β-catenin results in reduced expression of glioma-associated oncogene transcription factors, which are activated by HH signaling, indicating potential WNT regulation of HH signaling within the EHBD (31). Studies have also reported that genetic loss of Indian HH (the predominant HH ligand in EHBD) results in a loss of *Wnt7b* expression during bone development, indicating that HH signaling can also regulate WNT ligand expression (15, 53). Further studies are needed to interrogate a potential interplay between WNT and HH signaling in the cholangiocyte proliferative response.

Additionally, bile duct obstruction induces bile stasis, disrupting bile acid signaling and inducing biliary distension. Parsing out how these upstream effects result in induction of WNT and HH signaling is critical to defining the injury responses in the EHBD. While bile acids have been shown to induce proliferation in cholangiocytes, the role of mechanical distension in the regulation of bile acid metabolism and cell survival pathways is less understood (54, 55). Therefore, additional studies investigating the mechanical regulation of cholangiocytes may provide further insight into the cholangiocyte proliferative response to EHBD injury.

*WNT7B* and WNT target genes are upregulated in CCA, and inhibition of canonical WNT signaling reduces CCA tumor burden in rodent models (25). Using transcriptomic analysis of the TCGA database, we confirmed the upregulation of proliferation markers and WNT targets in human CCA (Supplemental Figure 9). We also showed that human cholangiocytes express WNT ligands in vitro, which can support organoid growth via activation of the canonical β-catenin–dependent pathway. Thus, our findings in mouse models appear to be directly relevant to human cholangiocyte biology.

In conclusion, this study provides comprehensive data on the transcriptional and functional changes in the EHBD following acute obstruction. We demonstrate that WNT signaling is upregulated after obstruction. Cholangiocytes appear to be the primary target for the proproliferative effect of WNT signaling, and inhibition of WNT ligand secretion in EHBDs attenuates the cholangiocyte proliferative response in vivo and in vitro. Our in vitro organoid and explant models revealed that cholangiocytes can regulate their proliferation by expressing WNT ligands and activating canonical WNT signaling. This work informs future studies focused on the regulation of the EHBD injury response and provides new knowledge about the context-dependent complexity of the WNT signaling pathway.

## Methods

### Sex as a biological variable

Both male and female mice were used for in vivo and transcriptomic investigation of the EHBD injury response. PCA of the bulk RNA-Seq (Figure 1D) demonstrated similar responses among male and female samples to bile duct injury. Despite not having seen variation attributed to sex in the PCA plots, we did include it as a term in our statistical analyses, thereby, ensuring that any consistent variation that was sex specific would not adversely affect the significance in our conditional comparisons. Organoids were derived from EHBD tissues of both male and female mouse and human tissue samples. Since organoid lines showed similar responses to WNT antagonism/activation across lines, data were combined for statistical analysis.

### Animal studies

#### Housing.

All animal studies were performed in accordance with approved protocols by the University of Michigan IACUC. Adult WT C57BL/6J (The Jackson Laboratory, stock no. 000664) mice were used for all studies. Mice were bred at the University of Michigan, housed in ventilated cages, with ad libitum access to food (Purina LabDiet, 5L0D) and water, and maintained on a 12-hour light/dark cycle. For experiments, mice were age- and sex-matched with littermate controls used whenever possible.

#### Surgeries.

BDL was performed as previously described (15). In brief, both sham and BDL mice underwent a laparotomy. For BDL in mice, the distal end of the EHBD was ligated near the duodenum to induce biliary obstruction. The abdominal wall and epidermal layer were sutured separately. EHBD tissue was collected 24, 48, and 72 hours after ligation.

#### WNT inhibition in vivo.

C59 (MedChemExpress [MCE], HY-15659), a PORCN inhibitor, was reconstituted in dimethyl sulfoxide (DMSO) at 100 mg/mL. Aliquots were diluted in 0.5% methylcellulose/0.1% Tween 80 and administered at 50 mg/kg via i.p. injection. C59 was administered every 12 hours, starting at 24 hours prior to surgeries and continuing until euthanasia.

#### Assessment of proliferation.

EdU (Lumiprobe, 10540) was administered for examination of cellular proliferation as previously described (15). EdU was prepared the day of administration at a concentration of 2.5 mg/mL and administered 3 hours prior to euthanasia at 12.5 mg/kg via i.p. injection.

### Mouse EHBD explant studies

EHBDs were excised from mice following euthanasia. Explants were washed in PBS, placed in a 12-well cell culture plate, and cultured in 2% FBS, 1% penicillin-streptomycin (Thermo Fisher Scientific, 15140122) and Advanced DMEM/F-12 (Thermo Fisher Scientific, 12634010) (2%FBS/DMEM/F-12). CHIR (ApexBio, A3011) stock solution was made by reconstituting CHIR to 10 mM in DMSO and storing at –20°C. CHIR (10 μM) or DMSO (vehicle) was diluted in 2%FBS/DMEM/F-12 and added to treatment and control wells, respectively. In total, 200 μM EdU (Lumiprobe, 10540) was added to the media for the full duration of an experiment for the quantification of cellular proliferation. Media were replaced at 24 hours. Forty-eight hours after treatment, explants were fixed and processed similar to freshly isolated EHBDs as stated below using a formalin-fixed, paraffin-embedded (FFPE) method as previously described (52).

### Biliary organoid studies

#### Mouse biliary organoid establishment.

EHBD mouse organoids were established as previously described with minor modifications (56). A 20-minute incubation in Liberase TL (MilliporeSigma, 5401020001) at room temperature was used for EHBD tissue dissociation followed by trituration through a 20 gauge needle (42). Dissociated cells and tissue fragments were pelleted and resuspended in 100–200 μL Matrigel (Corning Inc., 356237) and plated in 30–50 μL patties in a 24-well plate. Mouse organoid growth media (Table 1) was renewed every 2–3 days, and organoids were passaged every 5–7 days. In total, 50 ng/mL epidermal growth factor (EGF) and 100 ng/mL fibroblast growth factor 10 (FGF10) were added for the first 2 days of culture establishment.

#### Human biliary organoid establishment.

Normal biliary tissue samples were procured through the Gift of Life (Ann Arbor, Michigan, USA). Human EHBDs were dissociated similar to mouse biliary organoid establishment as described above. The resulting suspension was placed into human organoid growth media (Table 2) containing 100 ng/mL hepatocyte growth factor and 100 ng/mL FGF10 for the first 2 days.

#### Organoid growth assessment.

CellTiter-Glo 3D Cell Viability Assay (Promega, G9681) was used to assess organoid growth via luminescent analysis of adenosine triphosphate (ATP) levels according to the manufacturer’s protocol. Specifically, CellTiter-Glo reagent was diluted 1:1 with DMEM-F12, and 150 μL was added to the Matrigel domes in a 48-well plate containing organoids. The mixture was incubated for 30 minutes in the dark, following which 100 μL of the dissociated organoid mixture was transferred to a white opaque 96-well plate. Luminescence was read on a SpectraMax M5 (Molecular Devices) using an integration time of 500 ms. Organoid size was evaluated by measuring area using ImageJ (NIH) from images taken with a stereomicroscope (Olympus, SZX16) and digital microscope camera (Olympus, DP72) using cellSens software (Olympus).

#### Pharmacological WNT activation and inhibition in organoid studies.

CHIR was made fresh from 10 mM stock solutions by diluting to 3 μM in LWRN-free organoid growth media (Tables 1 and 2) for both mouse and human organoids. C59 (MCE) was reconstituted to 100 mM in DMSO and stored at –80°C. Working concentrations were made fresh by further diluting in LWRN-free organoid media (Tables 1 and 2) to 10 μM for mouse and human organoids. IWR-1 (MCE, HY-12238) stock solution was made by reconstituting IWR-1 at a concentration of 10 mM in DMSO and storing it at –80°C. Working concentrations were made fresh by diluting the stock to 5 μM in WNT-free organoid media for both mouse and human organoids.

### Generation of WNT7B conditioned media

Plasmid synthesis and viral packaging were performed by the University of Michigan Vector Core. For the cloning process, pLL CAG-msc-Pgk-GFP-a2 puro was digested using Nhel/Xhol. The mouse *Wnt7b* fragment was synthesized using Twist Biosciences platform and inserted into the cut vector using Gibson Assembly method. The *Wnt7b* insert was then confirmed by Sanger sequencing, and both the “empty” control vector (pLentilox-CAG-Empty-PKG-GFP/puro) and a WNT7B vector (pLentilox-CAG-mWNT7b-PKG-GFP/puro) were packaged into lentivirus by the core. L cells (ATCC CRL-2648) provided by the Michigan Medicine Translational Tissue Modeling Laboratory were cultured in 10% FBS, 1% penicillin-streptomycin, and DMEM (Thermo Fisher Scientific, 11965092). For lentiviral infection, cells were cultured in 10% FBS, DMEM with 8 µg/mL polybrene, and a 1:3 dilution of viral supernatant for approximately 24 hours, following which the cells were returned to their standard media. In total, 20 μg/mL puromycin was used to select transduced cells expressing the plasmid, including puromycin resistance.

#### WNT7B conditioned media.

Cells were plated at approximately 80% confluence and allowed to grow until they reached 100% confluence in a T-150 flask. Once at 100% confluence, the media were replaced with 30 mL of 20% FBS, 1% penicillin-streptomycin, and DMEM-F12 media. Media were collected and filtered through a 0.2 μm pore size filter (Thermo Scientific, 0974188) on the fourth day of incubation. Conditioned media were stored at 4°C for short-term storage (7–10 days) or –80°C for long-term storage. Conditioned media from cells transduced with the control vector were used as control conditioned media.

#### WNT7B effects on mouse organoids.

For analysis of WNT7B effects on organoid growth, we replaced DMEM-F12 with 75% WNT7B conditioned media from L cells. Media were made fresh at the start of the experiment.

### Histological assessment

#### IHC.

Preparation of tissues for histology was performed as previously described (52). FFPE tissues were sectioned at 4 μm. H&E kit (Vector, H-3502) was used according to the manufacturer’s protocol. Masson’s trichrome stain kit (Epredia, 87019) was used according to the manufacturer’s protocol. Slides were mounted using Permount (Thermo Fisher Scientific, SP15100). Immunofluorescence was performed as previously described with minor modifications (52). In brief, tissue sections were placed in boiling Citric Acid Based Antigen Unmasking Solution (Vector Labs, H-3300) for 15 minutes. Sections were incubated in blocking solution (1% BSA, catalog: A-420-10 Goldbio; 10% serum goat or donkey, catalog: G9023 Sigma-Aldrich and catalog: GTX73205 GeneTex, respectively; and 0.3% Triton X-100, catalog: BP151 Thermo Fisher Scientific) for 1 hour prior to incubation in the primary antibody overnight at 4°C, and a secondary antibody incubation was performed for 1 hour. Antibodies were diluted in blocking solution. Primary and secondary antibodies and their concentrations are included in Table 3. EdU staining was performed per the manufacturers protocol using the Click-iT EdU Alexa Fluor 488 Imaging Kit (Invitrogen, C10337).

#### Morphometric analysis.

Images were taken using a Nikon Eclipse E800 microscope (Nikon) and processed using NIS-Elements imaging software (Nikon). Representative images of approximately 5 high-power fields were taken for each sample at 20×–40× magnification. Researchers blinded to the treatment groups performed morphometric analysis using ImageJ Cell counter plug-in (V1.53f51, NIH). Cholangiocytes and stromal cells were determined by CK19^+^ and CK19^–^ staining, respectively, or histological examination of tissues.

#### Trichrome grading.

Images of the complete EHBD were taken for each sample. Two reviewers blinded to sample treatments graded the slides from 0 to 4, with 0 being no visible collagen and 4 being the most concentrated. Scores were averaged across samples and reviewers.

### Transcriptional analysis

EHBD RNA extraction was performed as previously described (52). Briefly, EHBD were isolated and placed in RNAlater (Invitrogen, AM7020) until RNA isolation. RNeasy plus micro kit (Qiagen, 74034) or RNeasy micro kit (Qiagen, 74004) with DNase treatment (Qiagen, 79254) were used according to manufacturer’s protocol. To extract RNA from organoids, media were aspirated from wells containing Matrigel, and 1 mL of cold PBS was used to dissolve Matrigel and resuspend cells. The cell resuspension was pelleted, PBS was aspirated, and 350 μL RLT + 3.5 μL beta-mercaptoethanol were added to each well. In total, 125 μL 100% EtOH was added to the lysis buffer prior to placing on the column. RNeasy mini kit (Qiagen, 74104) was then used according to the manufacturers protocol with a 15-minute DNase treatment.

#### qPCR.

cDNA was synthesized using iScript cDNA Synthesis kit (Bio-Rad, 1708891). Expression levels were analyzed from biliary tissue using iTaq Universal SYBR Green Supermix (Bio-Rad, 1725121). Primer sequences (Table 4) were designed using NCBI Blast and purchased through Integrated DNA Technologies.

#### ISH.

Analysis of *Wnt7b* in sham/BDL tissues was executed using the RNAscope 2.5 HD Assay Brown Kit (ACD Bio, 322310) and probe *Wnt7b* (ACD Bio, 401131) per manufacturer protocol. Hematoxylin was used for nuclear counterstaining.

#### Bulk RNA-Seq.

Library preparation and sequencing were conducted by the University of Michigan Advanced Genomics core as follows. RNA concentration and quality was assessed using the BioAnalyzer or TapeStation (Agilent). cDNA was synthesized using the SMART-Seq mRNA kit (Takara) from 4 ng of RNA according to manufacturer’s protocols. Final libraries were checked for quality and quantity by Qubit hsDNA (Thermo Fisher Scientific) and LabChip (Perkin Elmer). A 151 bp paired-end sequencing was performed according to the manufacturer’s protocol (Illumina NovaSeq). BCL Convert Conversion Software v3.9.3 (Illumina) was used to generate demultiplexed Fastq files. Salmon (57) was used to count the trimmed data against the transcriptome defined in Gencode vM33 (58), which was built on the genome GRCm39. Several Bioconductor (v3.17) packages in R (v4.31) were used for subsequent steps. The transcriptome count data were annotated and summarized to the gene level with tximeta (59) and further annotated with biomaRt (60). PCA plots were generated with PCAtools (61), and principal component loadings were plotted in Graphpad Prism (v.10). Normalizations and statistical analyses were done with DESeq2 (62). Genes with very low expression (fewer than 10 counts) were removed. EnhancedVolcano (63), pheatmap (64), and ggplot2 were used to generate plots. Metascape (36) (v3.5) was used for gene ontology analysis.

#### scRNA-Seq analysis.

EHBDs were isolated from sham/BDL mice 24 hours after surgery. Tissues were isolated from 5–6 WT mice and rinsed in 1× cold phosphate buffered saline, followed by fixation and dissociation using the 10X Genomics Next GEM Single Cell Fixed RNA Sample Preparation Kit. Two cell preparations from sham and BDL (1 male, 1 female; each preparation included 5 mice) were performed. Fixed cells were stored at –80°C prior to library preparation. Library preparation and sequencing was conducted by the University of Michigan Advanced Genomics Core. Library preparation was performed according to 10X Genomics protocols using the Chromium Mouse Transcriptome Probe Set v1.0.1, which is derived from the mm10-2020A transcriptome. A 151 bp paired-end sequencing was performed according to the manufacturer’s protocol (Illumina NovaSeq). BCL Convert Conversion Software v3.9.3 (Illumina) was used to generate demultiplexed Fastq files. Outputs were input into CellRanger (v7.1.0) to perform alignment, filtering, barcode counting, and UMI counting and to construct feature-barcode matrices. The Seurat (version4.1.1) package (65) was used to filter, integrate, normalize, and cluster the data. H5 files from the cell ranger output were imported for each sample. Samples were filtered to include cells that showed expression of between 200 and 9,000 genes and less than 5% mitochondrial genes. Expression data were normalized with the sctransform (v2) method and integrated using the RPCA method. The integrated dataset was clustered with a resolution of 0.3, yielding 21 clusters. Both 2D and 3D Uniform Manifold Approximation and Projections (UMAPs) were calculated. The Seurat object was exported and processed for visualization and further exploration using Cirrocumulus (66). Bubble plots in Seurat were used to assess gene expression with cell clusters. Characterization of cell clusters were identified by comparing DEGs broadly expressed within the cluster to The Human Protein Atlas.

#### Liver function tests.

Serum was collected from mice immediately following euthanasia via a cardiac puncture. Blood was allowed to coagulate for 30 minutes, followed by centrifugation at 3,000*g* to separate the serum. Liver chemistries were run by the University of Michigan ULAM Pathology Core.

### Statistics

One-way ANOVA with Bonferroni’s multiple-comparison test, 2-way ANOVA with a Bonferroni’s multiple-comparison test, unpaired 2-tailed Student’s *t* test, 2-tailed 1-sample *t* test, and Fisher’s exact test were used to assess statistical significance as indicated. *P* < 0.05 was considered significant. All analyses were performed in GraphPad Prism V.10. Data are represented as ± SEM.

### Study approval

All mouse studies were performed under the University of Michigan IACUC–approved protocol PRO00011285.

### Data availability

Twenty-four– and 48-hour bulk RNA-Seq datasets are deposited in the NCBI Gene Expression Omnibus (GEO) database under accession nos. GSE280724 and GSE280691, respectively. scRNA-Seq datasets are deposited under accession no. GSE280889. Values for all data points in graphs are reported in the Supporting Data Values file.

## Author contributions

ANC, NR, and LCS were responsible for study design. ANC, NR, MQP, NHMZ, and TMK conducted all experiments. NR and TLF were responsible for sample procurement. ANC, JWT, and NR were responsible for data analysis. ANC and NR wrote the manuscript, and all other authors provided comments on the manuscript.

## Supplementary Material

Supplemental data

Supplemental table 1

Supporting data values

## Figures and Tables

**Table 4:**
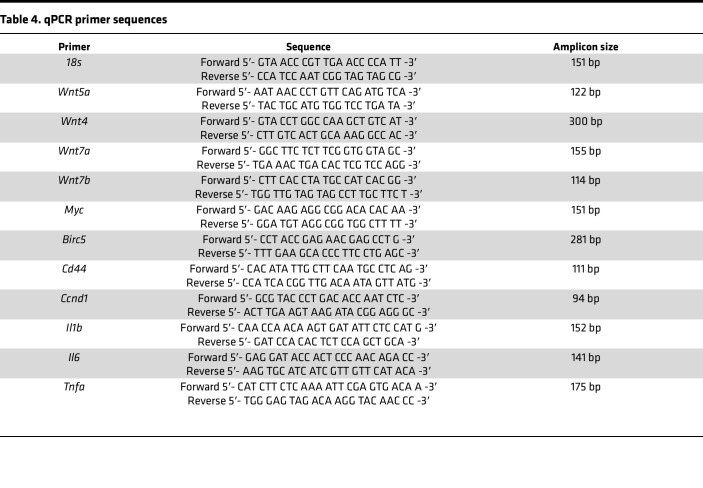
qPCR primer sequences

**Table 3:**
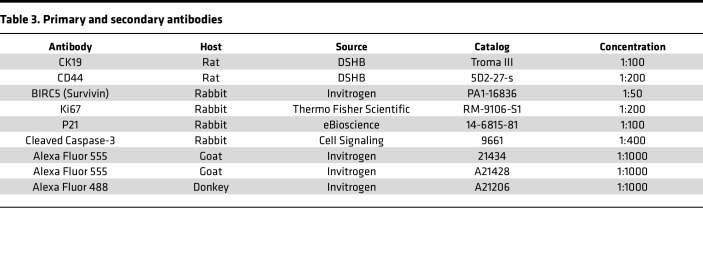
Primary and secondary antibodies

**Table 2:**
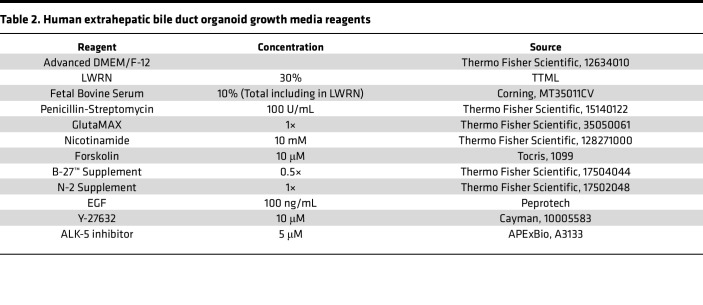
Human extrahepatic bile duct organoid growth media reagents

**Table 1:**
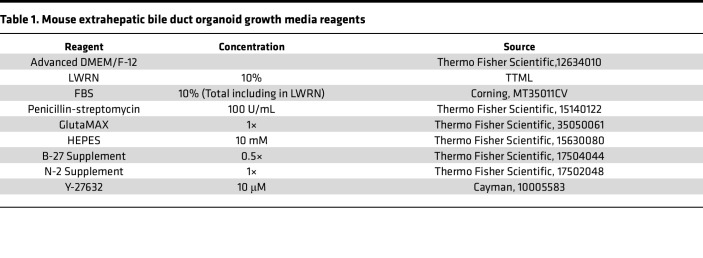
Mouse extrahepatic bile duct organoid growth media reagents

## Version 1 12/05/2024

In-Press Preview

## Version 2 01/23/2025

Electronic publication
