# Supplementary material for: WNT signaling contributes to the extrahepatic bile duct proliferative response to obstruction in mice
Source: JCI Insight. 2024 Dec 5;10(2):e181857. doi: 10.1172/jci.insight.181857 (PMC11790017; doi:10.1172/jci.insight.181857)
Supplement: Supplemental data [file jciinsight-10-181857-s167.pdf]

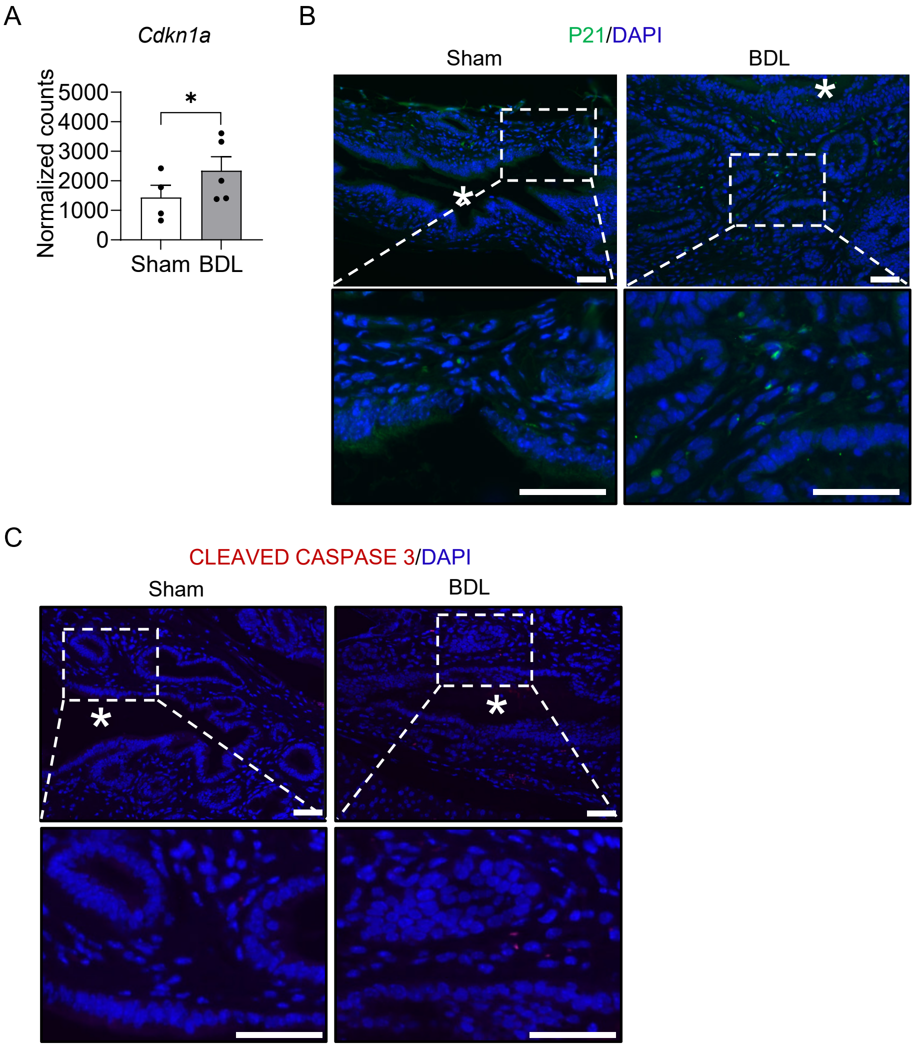

Supplemental Figure 1: Cell senescence and apoptosis at 24-hours post-injury. A) *Cdkn1a* (gene encoding P21) expression in sham/BDL mice from bulk RNA-seq dataset at 24-hours post-surgery. n=4-5 mice/group. **B)** P21 immunofluorescence localizes P21 primarily to the stromal cell compartment. **C)** An absence of apoptotic cells is shown by cleaved caspase 3 staining in the EHBD. Scale bar: 50 µm. P-value: *<0.05. Immunofluorescence: n=3-4 mice/group.

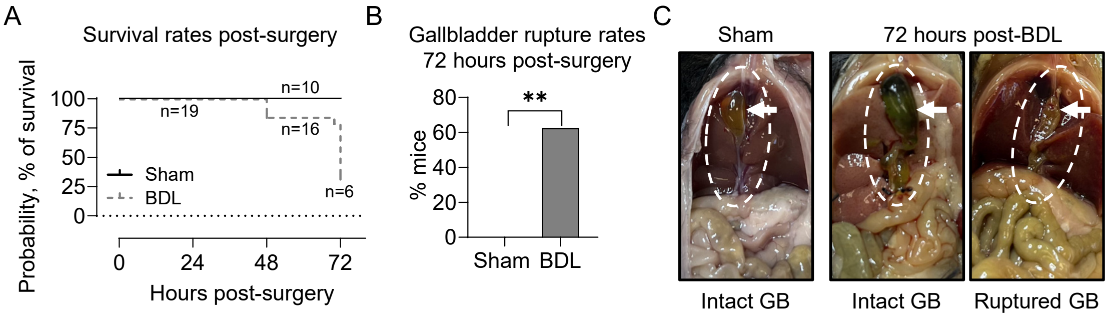

Supplemental Figure 2: Complete distal extrahepatic bile duct obstruction leads to high mortality rates within 72-hours post-surgery. A) Mortality rates in sham/BDL mouse cohorts by 72-hours. n=10-19 mice/group. **B)** Gallbladder status in mice that survived to the 72-hour timepoint. **C)** Images from mice 72 hours post-surgery with intact and ruptured gallbladders. Fisher's exact test was used to assess statistical significance. P-value: **<0.01.

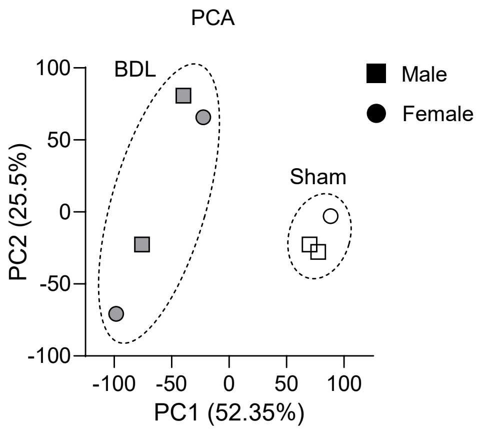

Supplemental Figure 3: Principal component analysis from bulk RNA-seq of the mouse EHBD 48 hours post-surgery. n=3-4 mice/group.

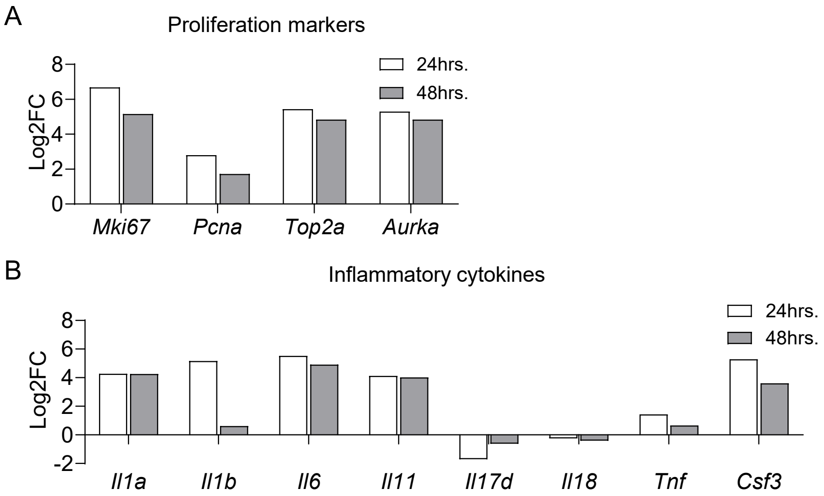

Supplemental Figure 4: Proliferation and inflammatory genes expression at 24- and 48-hours post-BDL. Analysis of bulk RNA-seq datasets at 24- and 48- hours post-BDL for **A)** proliferation and **B)** inflammatory markers in mice. Log2FC of BDL to sham mice. n=3-5 mice/group.

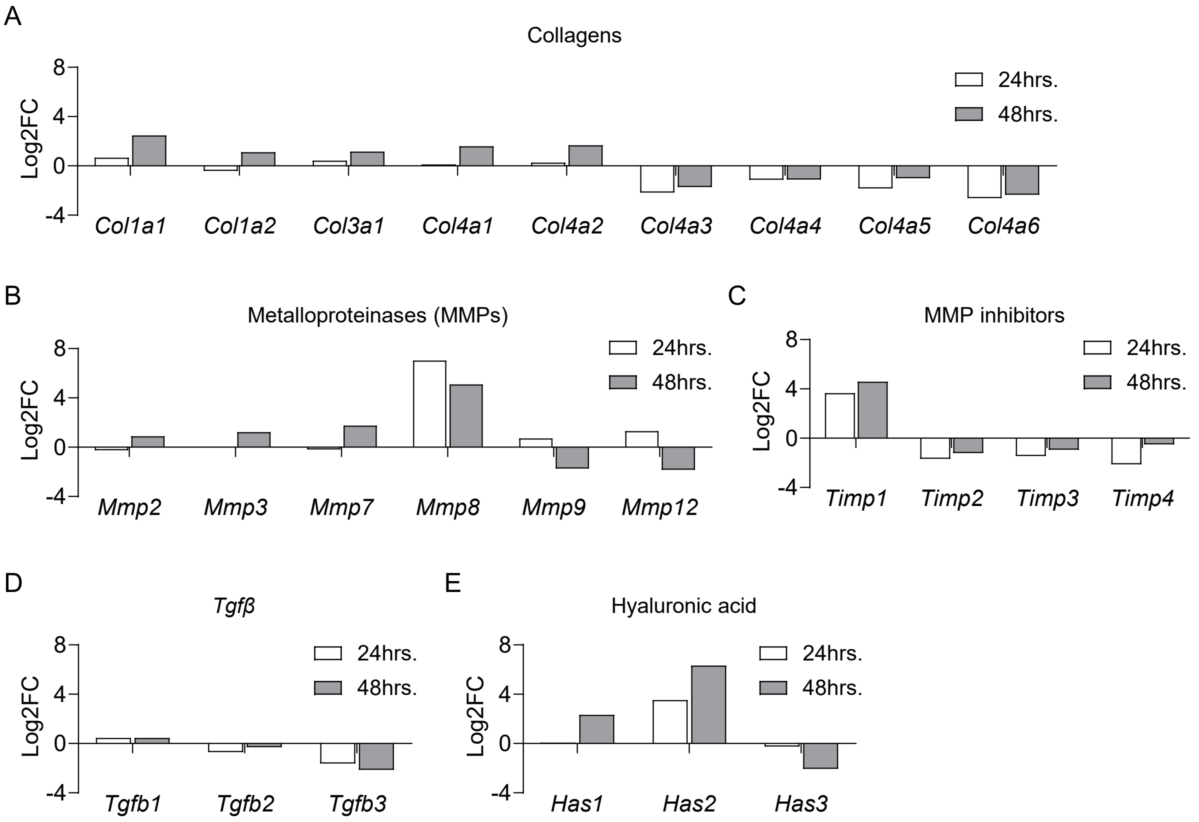

Supplemental Figure 5: Transcriptional changes in extracellular matrix related markers 24 and 48-hours after BDL. Bulk RNA-seq analysis of extracellular matrix related markers. **A)** collagens, **B)** metalloproteinases (MMPS), **C)** MMP inhibitors **D)** *Tgfβ* and **E)** Hyaluronic acid genes. Log2FC of BDL to sham mice. n=3-5 mice/group.

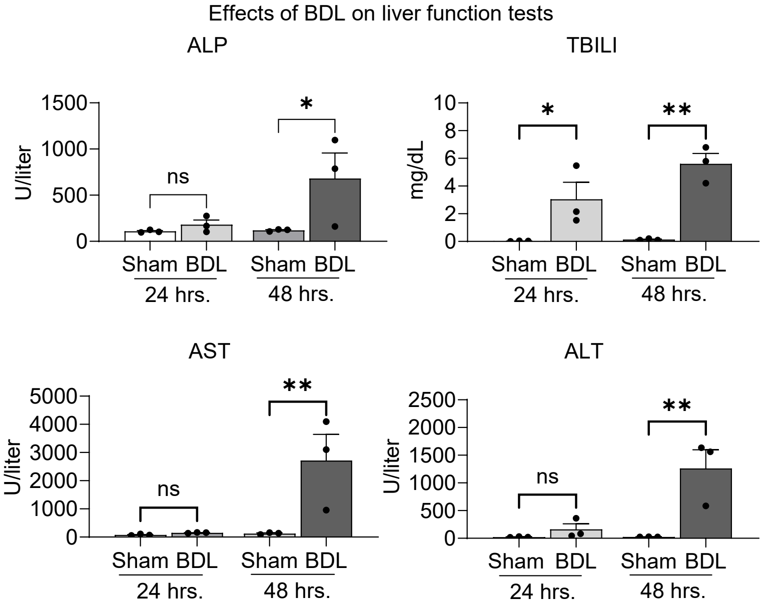

Supplemental Figure 6: Effects bile duct ligation on liver function tests following 24 and 48-hour sham/BDL surgeries. Analysis of serum levels following 24- or 48-hour sham/BDL surgeries. Statistical significance was assessed by a one-way ANOVA with Bonferroni's multiple comparison’s test. P-value: *<0.05, **<0.01, ns=not significant. n=3 mice/group.

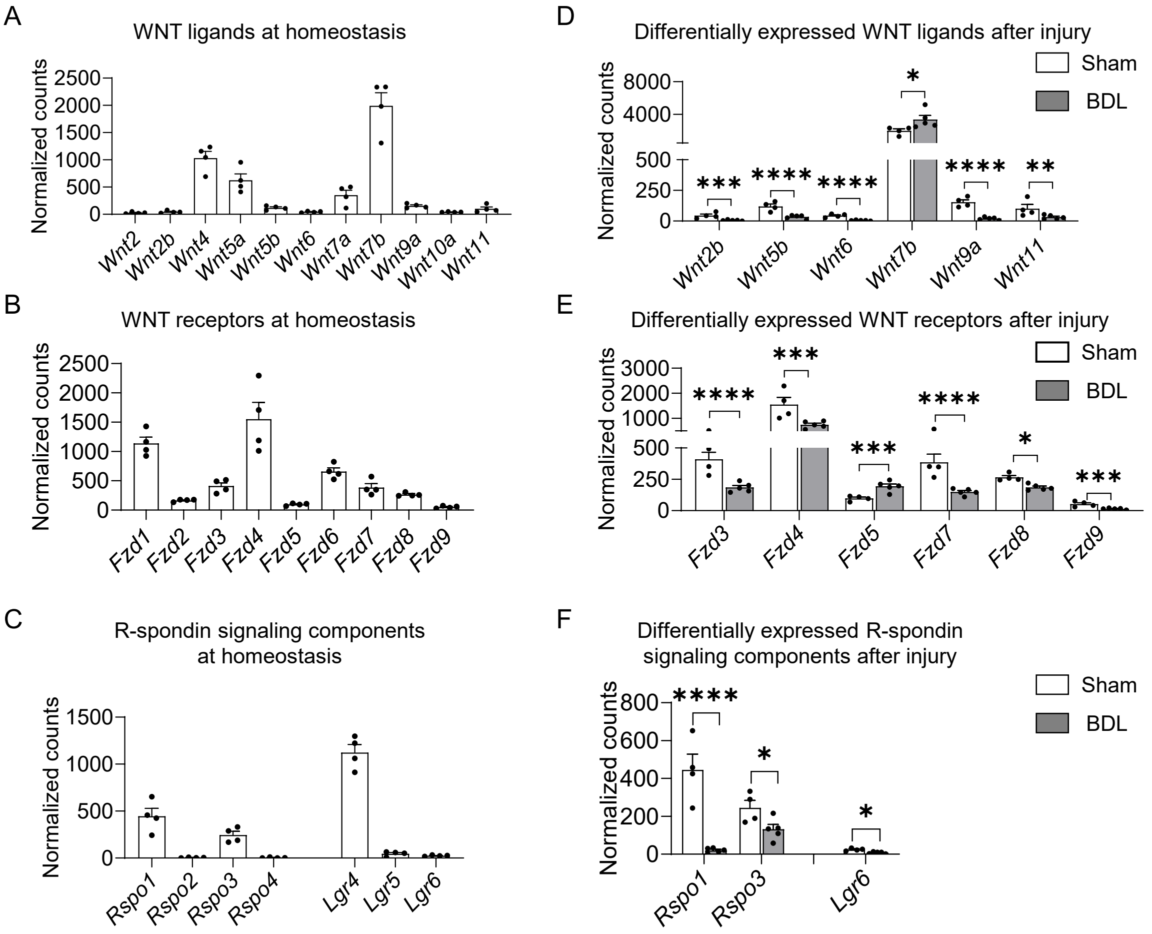

Supplemental Figure 7: WNT ligand and receptor expression in the mouse EHBD at homeostasis and 24-hours after injury. A-C) WNT ligands, receptors, and potentiators (R-spondin's) expression from bulk RNA-seq 24 hours post-sham. **D-F)** Expression of all differentially expressed WNT ligands, receptors, and R-spondin's in sham/BDL mice. Statistical significance of bulk RNA-seq was assessed using DESeq2. P-value: *<0.05, **<0.01, ***<0.001, ****<0.0001. n=4-5 mice/group.

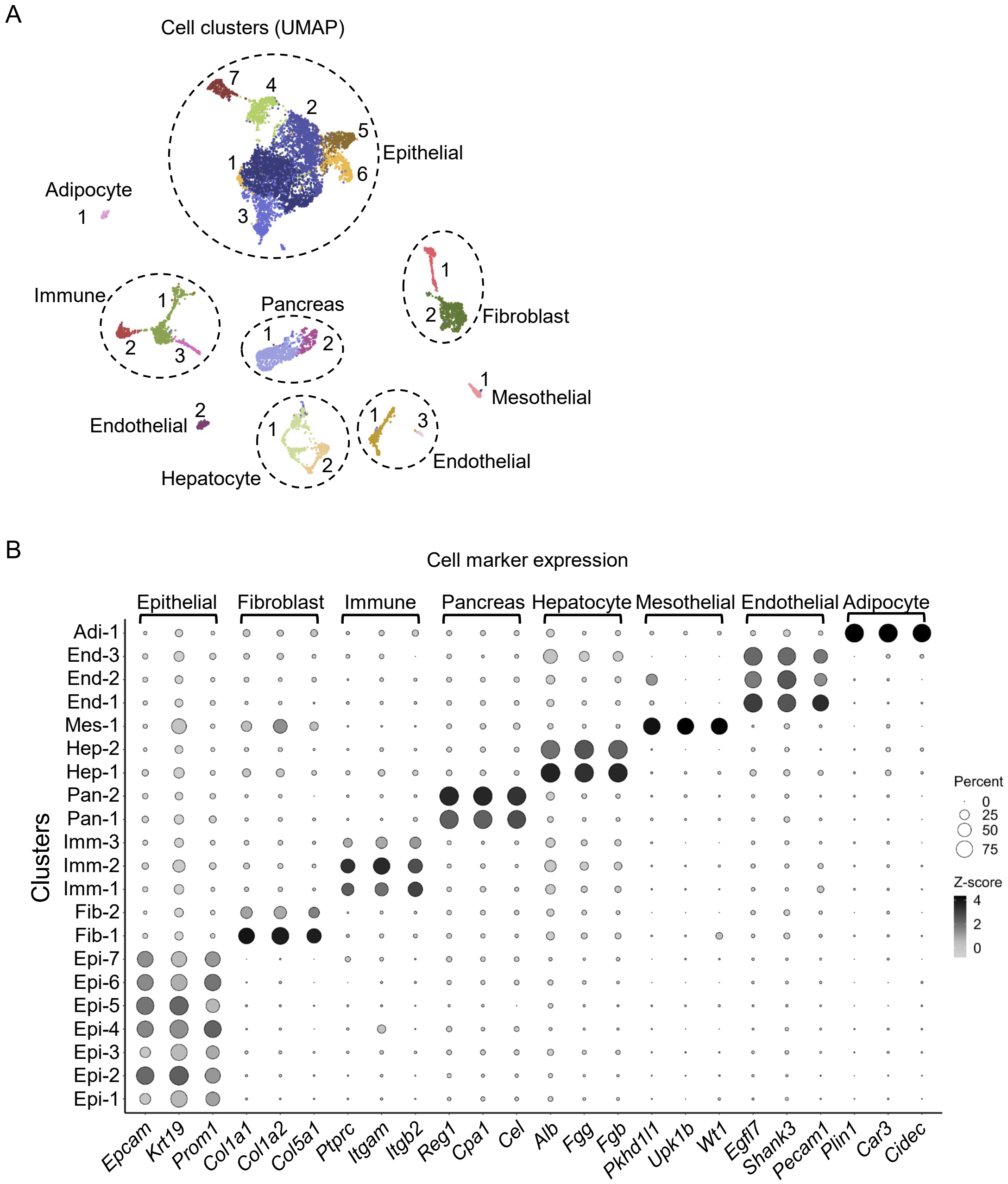

Supplemental Figure 8: Single-cell RNA-seq cell clusters and markers. A) UMAP showing 21 unique cell clusters as determined using Seurat. **B)** Marker genes (x-axis) used to define cell clusters; Seurat clusters (y-axis) relabeled dependent on predominant marker genes in that cluster. n=2 samples/treatment, n=5 mice/sample.

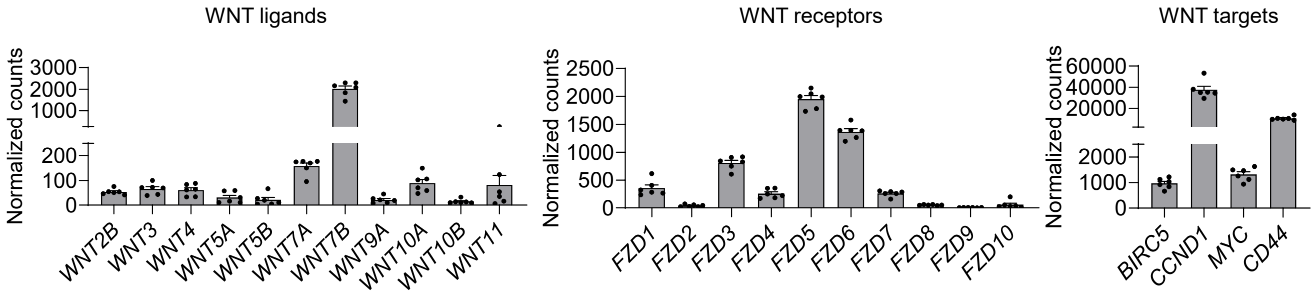

Supplemental Figure 9: Human EHBD cholangiocytes express WNT ligands and are WNT target cells. WNT ligand and receptor expression in human EHBD organoids based on the published bulk RNA-seq dataset (E-MTAB-7569). n=3-6 samples/group.

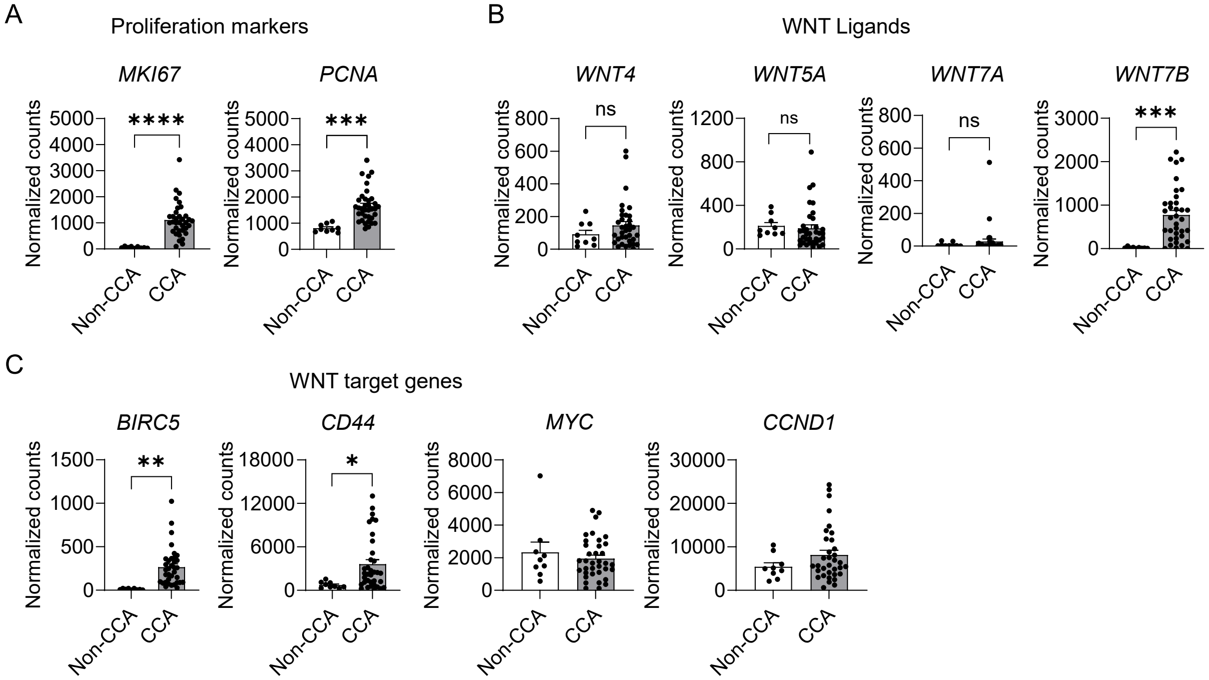

Supplemental Figure 10: Human CCA is associated with increased cellular proliferation and WNT signaling. A) Proliferation markers **B)** WNT ligands and **C)** WNT target gene expression in cholangiocarcinoma (CCA) samples analyzed from the TCGA database. Outliers +/- 3 SD from the mean were removed. Unpaired student's *t*-test. P-value: *<0.05, **<0.01, ***<0.001, ****<0.0001. n=9-36 samples/group.

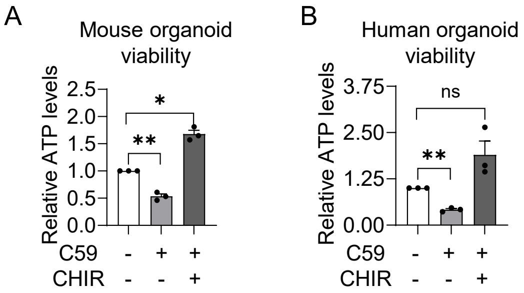

Supplemental Figure 11: Canonical WNT activation rescues organoid growth following inhibition of WNT ligand secretion. A) mouse organoids **B)** human organoids treated with C59 (10 μM) to inhibit WNT ligand secretion, CHIR (5 μM) to activate canonical WNT signaling, or both. One-sample *t*-test. P-value: *<0.05, **<0.01, ns=not significant. n=3 biological replicates.

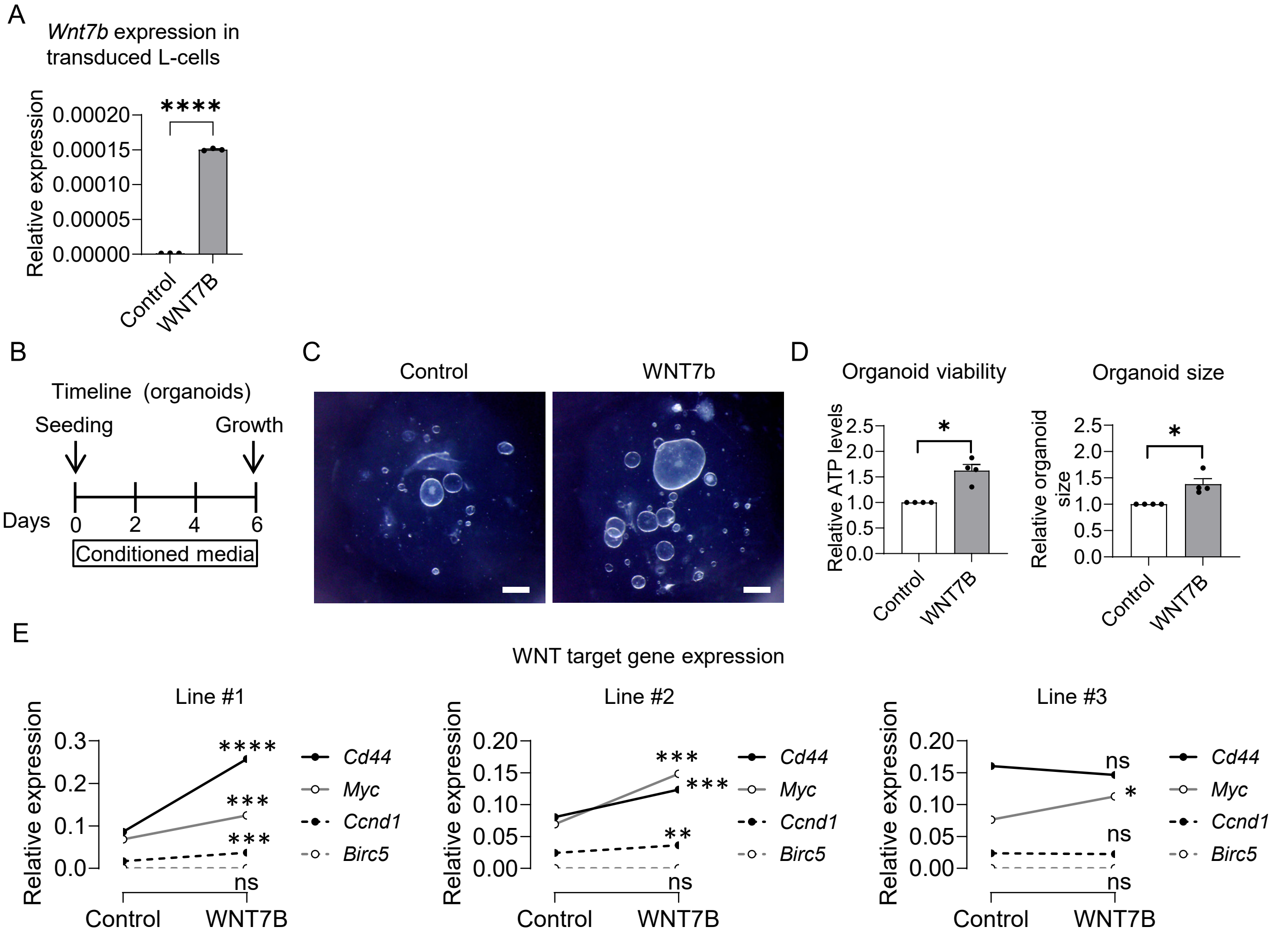

Supplemental Figure 12: WNT7B enhances organoid growth and WNT target gene expression in mouse EHBD organoids. A) *Wnt7b* expression from L-cells were transduced with a control (lacking *Wnt7b*) vector or a vector containing *Wnt7b*. n=3 technical replicates. **B)** Timeline of mouse organoid experiment for size and ATP measurements. **C)** Images of organoids treated with control media (conditioned media from L-cells without induced WNT7B) and WNT7B conditioned media. **D)** Organoid growth and size measurements in control and WNT7B treated organoids following 6 days of treatment. Outliers +/- 3 SD from the mean were removed. Scale bar: 500 µm. One-sample *t*-test. P-value: *<0.05. n=3 biological replicates. **E)** Relative gene expression levels in three different organoid lines following 24-hour treatment in WNT7B conditioned media. n=3 technical replicates. Unpaired student's *t*-test. P-value: *<0.05, **<0.01, ***<0.001, ****< 0.0001, ns=not significant.

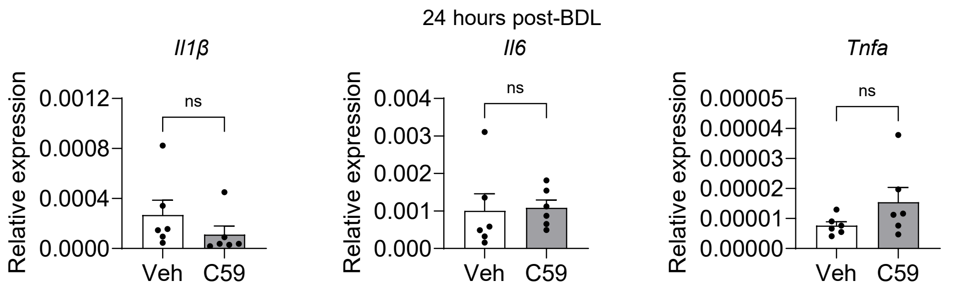

Supplemental Figure 13: WNT inhibition does not affect inflammatory genes at 24-hours post-BDL. C59 treated mice show similar expression levels of inflammatory markers at 24-hours post-BDL to vehicle controls. Student's *t*-test was used to assess statistical significance. ns=not significant. n=6 mice/group.

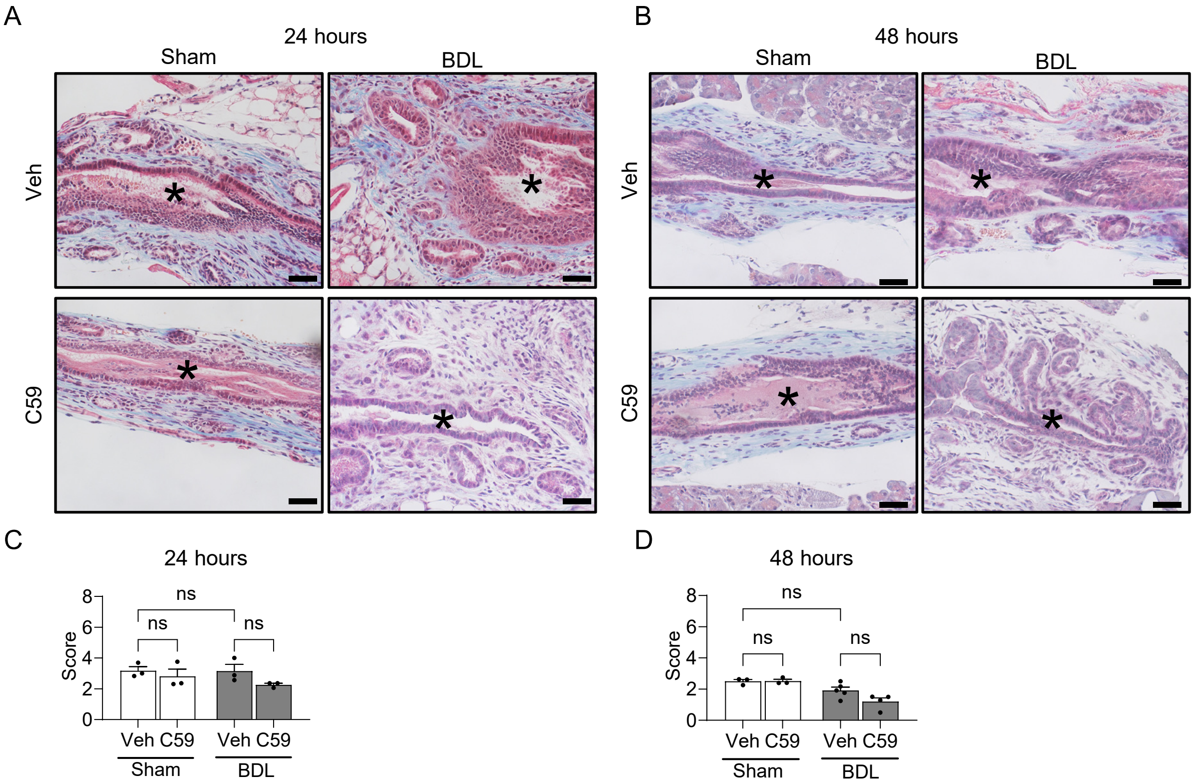

Supplemental Figure 14: WNT inhibition did not alter collagen expression in mouse EHBDs 24- and 48- hours post-surgery. Masson trichrome staining on EHBDs at **A)** 24-hours and **B)** 48-hours after sham/BDL surgeries. Grading of trichrome staining from **C)** 24- and **D)** 48-hours post-surgery. 2-way ANOVA with Bonferroni's multiple comparison's test was used for statistical analysis. ns=not significant. Scale bar: 50 μM. n=3-5 mice/group.

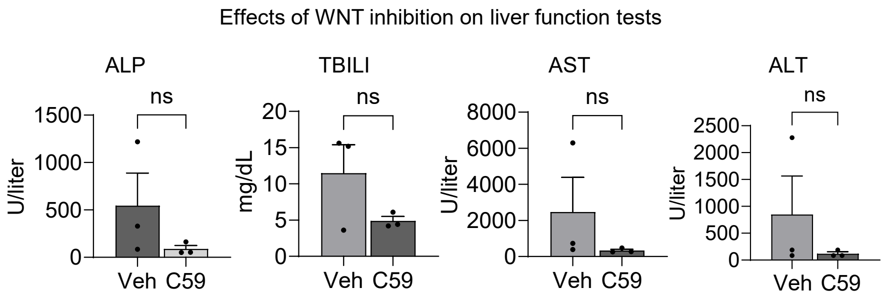

Supplemental Figure 15: C59 did not change liver function tests following 48-hour BDL. Serum levels of C59 and Veh treated 48-hour sham/BDL mice. Statistical significance was assessed by a student's t-test. P-value: ns=not significant. n=3 mice/group.
